# Considerations for Studying Sex as a Biological Variable in Spinal Cord Injury

**DOI:** 10.3389/fneur.2020.00802

**Published:** 2020-08-05

**Authors:** Andrew N. Stewart, Steven M. MacLean, Arnold J. Stromberg, Jessica P. Whelan, William M. Bailey, John C. Gensel, Melinda E. Wilson

**Affiliations:** ^1^Department of Physiology, University of Kentucky, Lexington, KY, United States; ^2^Spinal Cord and Brain Injury Research Center, College of Medicine, University of Kentucky, Lexington, KY, United States; ^3^Department of Statistics, College of Arts and Sciences, University of Kentucky, Lexington, KY, United States

**Keywords:** gender, stroke, traumatic brain injury (TBI), estrogen, testosterone, bladder, pain

## Abstract

In response to NIH initiatives to investigate sex as a biological variable in preclinical animal studies, researchers have increased their focus on male and female differences in neurotrauma. Inclusion of both sexes when modeling neurotrauma is leading to the identification of novel areas for therapeutic and scientific exploitation. Here, we review the organizational and activational effects of sex hormones on recovery from injury and how these changes impact the long-term health of spinal cord injury (SCI) patients. When determining how sex affects SCI it remains imperative to expand outcomes beyond locomotor recovery and consider other complications plaguing the quality of life of patients with SCI. Interestingly, the SCI field predominately utilizes female rodents for basic science research which contrasts most other male-biased research fields. We discuss the unique caveats this creates to the translatability of preclinical research in the SCI field. We also review current clinical and preclinical data examining sex as biological variable in SCI. Further, we report how technical considerations such as housing, size, care management, and age, confound the interpretation of sex-specific effects in animal studies of SCI. We have uncovered novel findings regarding how age differentially affects mortality and injury-induced anemia in males and females after SCI, and further identified estrus cycle dysfunction in mice after injury. Emerging concepts underlying sexually dimorphic responses to therapy are also discussed. Through a combination of literature review and primary research observations we present a practical guide for considering and incorporating sex as biological variable in preclinical neurotrauma studies.

## Introduction

In most areas of scientific study, knowledge gained from both pre-clinical and clinical research is based upon a disproportionate inclusion of male subjects. Implications of this male-dominated research are that guidelines developed from medical literature often neglect sex-based differences in basic pathophysiology of disease and treatment responses. Modeling medical practice on such limited demographics and failure to advance our understanding of disease, injury, and treatment in the context of sex-based differences have manifested into practices that are emerging as not just ineffective, but sometimes dangerous, to the health of women. For these reasons, in 2015 the National Institute of Health (NIH) has announced the expectation that “*scientists will account for the possible role of sex as a biological variable in vertebrate and human studies*” (1). In recent years, likely owing to this mandate, findings from animal models of many neurological conditions have begun exposing exactly how important sex-dependent effects in medicine can be. This manuscript evaluates work that has considered sex as a biological variable in neurotrauma with specific emphasis on spinal cord injury (SCI). Further, we provide novel primary data demonstrating that sex effects in SCI can depend on age at time of injury. Because pre-clinical work comparing male and female responses to SCI is limited, outcomes are frequently paralleled to findings in traumatic brain injury (TBI). A recent and more thorough review of sex effects on TBI can be found elsewhere (2). Finally, methodological considerations for assimilating sex as a biological variable in SCI studies are discussed owing to a substantial increase in the complexity of study design and interpretation.

## Materials and Methods

Materials and methods used to construct **Figures 2**–**5** have been provided in Supplementary Materials. Data provided in Figures 2–5 is primary data used to articulate sex-dependent relationships important for the consideration of studying sex in pre-clinical models of SCI.

## Results and Discussion

### Clinical Observations Support That Females Recover Better Following Neurotrauma

The first observations of sex differences in neurotrauma found that men experience a higher frequency of cerebral infarcts (3) and increased mortality compared to women (4, 5). Meta-analyses of clinical data in SCI patients have found mixed results, with a tendency for females to experience improved recovery compared to male counterparts in measures of motor capabilities and independence (6). Differences in demographic characteristics between males and females, however, introduce several caveats that complicate the interpretation of how sex affects SCI recovery. Historic incidence rates of SCI disproportionately affect males, with over 80% of SCI occurring in males between 25 and 45 years of age (7). In contrast, on average females tend to receive SCI at an older age (8, 9). Older age at time of SCI can exacerbate injuries (10–12) and mechanisms of primary trauma at older ages are often caused by less forceful events such as slip and fall accidents compared to vehicular and sporting accidents or acts of violence (13). However, even when age is controlled, in the clinical setting, females recover better than males (6). Finally, emergent work in animal models has also reproduced a small but significant protective effect of being female following SCI (14–16).

#### Pre-clinical Data Indicate That Sex-Differences Are Outcome Specific

The extent to which sex influences outcomes following SCI remains controversial based on existing clinical and pre-clinical data. Several rodent studies have confirmed a female-biased protection on locomotor outcomes after SCI (14–17), while others have found no differences (18, 19). Most prior work supporting sex-dependent effects after SCI have limited evaluations to locomotor outcomes and white matter sparing, which found marginal improvements favoring females. However, problems facing patients suffering from SCI extend beyond an inability to walk. Most patients suffering from thoracic/lumbar SCI report the largest depreciation in quality of life arising from secondary complications such as developing neuropathic pain (20), urinary and bowel incontinence (21), as well as sexual dysfunction (22), rather than an inability to walk. Following cervical SCI, which makes up 54.5% of all reported SCI conditions (13), disability is expanded to dysfunction of upper limbs and potentially to respiratory control, both of which further depreciate quality of life after injury (23). Indeed, relieving these secondary complications is of highest priority for individuals with SCI (24). Therefore, it is necessary to expand pre-clinical outcomes beyond locomotor disability to determine if sex differences exist in other modalities of SCI-induced dysfunction and to understand what underlying biological processes mediate these effects.

Unlike reports of locomotor functions, clinical reports suggest that no differences exist between males and females in the development or severity of bowel or bladder incontinence (25), or in the frequency of developing urinary tract infections (26). However, females do have a higher clinical incidence for reporting development of SCI-induced pain (27, 28). What little work has been done in animal models to compare a sex-dependency of pain development after SCI has also demonstrated controversial results. Female rats have been reported to both increase (29) and decrease (30) the prevalence of developing mechanical and thermal allodynia after SCI, while no sex-dependent effects have been found in mice (31, 32). Importantly, several studies investigating analgesic strategies to reduce pain caused by peripheral nerve injury have converging evidence that many pain-relieving agents exert sexually dichotomous effects (33–38). A similar sex-dependent effect was found using pioglitazone to treat SCI-induced pain in mice which found a female-specific analgesic influence (31). These findings suggest that while the experience of SCI-induced pain may not differ between sexes in mice, biological mechanisms regulating pain may differ between males and females. Extrapolating these findings to other outcomes may suggest that despite small sex-dependent effects in outcomes of locomotion or pain, the biological mechanisms underlying dysfunction may differ and require different strategies for treatment.

### Female Sex Hormones Are Potentially Neuroprotective

The investigation of sex-specific effects in animal models of neurotrauma has predominately focused on how sex hormones mediate tissue protection (39). Due to a higher prevalence and fluctuation of estrogens and progesterone in females, it is reasonable to hypothesize that female sex hormones are neuroprotective. Two major design strategies have been employed to support this hypothesis *in vivo* following neurotrauma. These include ovariectomies to partially deplete estrogens and progesterone, as well as exogenous delivery of estrogens and progesterone in both female and male rodents prior to injury (5, 39–41). Ovariectomies normalize tissue and functional outcomes between sexes, a finding consistent following both TBI (40) and SCI (41). This supports female sex hormones as being modestly neuroprotective. Using estrogens or high-dose progesterone as treatments for neurotrauma has persistently improved outcomes following SCI, TBI, and stroke in both males and females (5, 39, 42–48). The influence of female hormones on recovery from neurotrauma has led to an appraisal that inclusion of females adds too much variability to data due to the fluctuation of estrogens and progesterone during the estrus cycle, which scientists use as an argument to exclude the use of females in most pre-clinical research.

### Females Persist as the Predominate Sex Used in Pre-clinical Studies of SCI

**Figure 1:**
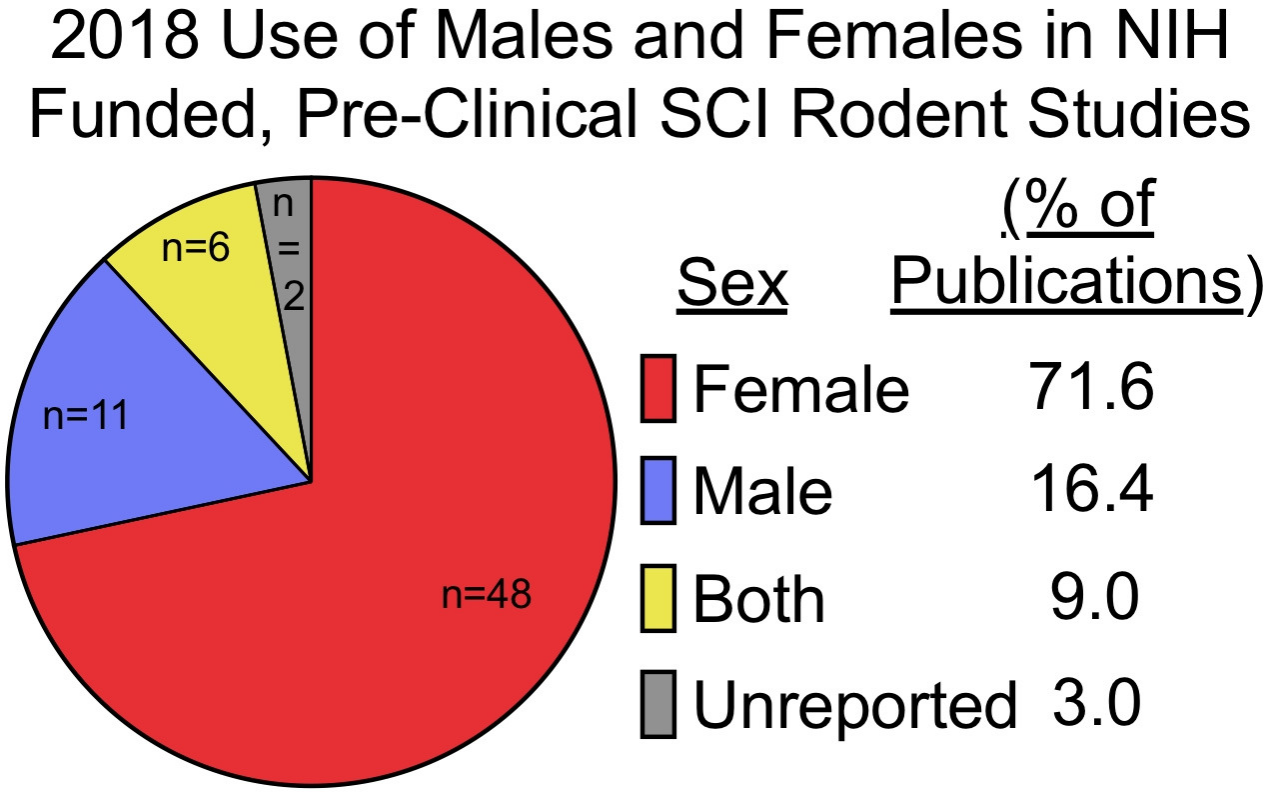
Females are used exclusively in most pre-clinical SCI research funded by the NIH. Pre-clinical, rodent, primary literature research papers funded by the NIH, published in 2018, and publicly available through Pubmed Central were analyzed for inclusion of sex as a biological variable (*n* = 67; published studies analyzed are available in Supplementary Table 1). Females (*n* = 48) were the predominate sole sex used, followed by males (*n* = 11), both (*n* = 6), and unreported (*n* = 2). Of studies utilizing both male and female rodents, only one study explicitly reported on how data between sexes were compared and included in analysis (32). Search function included: [(rat) OR mouse] AND [(((Spinal cord injury[Title]) OR spinal cord contusion[Title]) OR spinal cord transection[Title]) AND (“2018/01/01”[PDat]: “2018/12/31”[PDat])].

A belief that hormonal fluctuations during the estrous cycle adds variability to research outcomes is contributing to the exclusion of females in most pre-clinical neurotrauma modeling. However, contrary to the TBI and stroke fields, female rodents are the preferred sex to model SCI. Data analyses of NIH-funded, rodent, primary research publications demonstrate that females are the sole sex used in the vast majority of SCI experiments (Figure 1). This may change, as our data (compiled from freely available 2018 publications), likely does not yet reflect NIH programmatic changes enacted in 2016 to consider sex as a biological variable in vertebrate animal research. Nonetheless, male rodents are not often used when modeling SCI due to more severe post-operative complications and difficulty with manual bladder expressions which are required after experimental paralysis. These severe, male-specific, postoperative complications confound research efforts by increasing mortality and exclusion of subjects due to adverse health issues. A bias against male rodents in pre-clinical models of SCI has created a unique incongruence between clinical and pre-clinical demographics because the predominant clinical demographic is young males. In fact, the smallest SCI demographics seen in clinic are young and elderly females (8, 9, 49). This would argue that even if females were to be used, middle-aged female rodents would serve as a more clinically translatable model. Considering that neither young males, nor middle-aged females are commonly used to model SCI, including these additional variables may be essential for improving translatability of pre-clinical findings.

The importance of including both sexes in pre-clinical SCI research is emphasized by findings that support sex-dependent effects in both locomotor (15, 16), and non-locomotor outcomes such as pain (29, 30). An accumulation of recent work is finding that the pathophysiology of injury is fundamentally different between males and females (50, 51). Similarly, males and females have sex-specific considerations for long-term care (27, 52), and biological differences can alter response to treatment (31, 35, 53). The rest of this manuscript will discuss how several physiological processes differ between males and females and highlight how these differences affect injury, recovery, and living with SCI.

### Organizational and Activational Effects of Sex Hormones in SCI

#### Perinatal Development Induces Lasting Organizational Differences on Neuroanatomy, Cell Distribution, and Epigenetic Profiles

As mentioned above for sex hormones, the investigation of sex-specific effects in animal models of neurotrauma has predominately focused on activational changes. These transient effects on hormone levels throughout life, or “activated” in response to injury, influence secondary injury cascades, inflammation, and repair after SCI as discussed in more detail below. However, sex-specific organizational effects, those that occur during development and throughout life, shape the nervous system at a structural and cellular level and contribute to sex differences in behavior and functional responses (54). In the brain neuronal cell numbers in discrete areas differ between males and females which are established during the organizational period of hormone exposure (55). Similar sex differences in astrocytes and microglia cell numbers and morphology have been reported (55). However, little has been performed evaluating sex differences in the spinal cord outside of the regulatory centers controlling male and female sex organs (56). These neuroanatomical differences are hypothesized to be mediated by perinatal exposure to steroids, specifically a prenatal surge of testosterone occurring in males late in gestation and a second surge occurring immediately after birth (57, 58).

One example of sex differences arising during spinal cord development is observed in the spinal nucleus of the bulbocavernosus (SNB); a pool of motoneurons in the lower lumbar spinal cord. SNB motoneurons project to striated muscles of the perineum which attach to the base of the penis and are required for an erection and ejaculation (59, 60). Male rats have more cells in the SNB compared to females (59). Developmentally, SNB motoneurons initially form in both sexes but degenerate in females around the time of birth (56). In addition to the neurotrophic signals from the muscles, androgens and estrogens have been shown to permanently establish this sex difference early in development. The lumbar spinal cord houses neurons and central pattern generators controlling functions that are disrupted by SCI including pain responses as well as locomotor, bowel, and bladder functions. Whether other organizational effects exist within the spinal cord and contribute to sex-specific injury responses remain to be determined.

Organizational effects during development also confer innate sex-differences in epigenetic profiles and cell morphology that do not depend on ongoing sex hormone signaling (61, 62). For example, early developmental exposure to sex hormones is thought to induce a permanent sexual phenotype of glial cells (62), which recently has been demonstrated in brain microglia (63). Microglia display a sex-dependent morphology, with female microglia having a more ramified morphology compared to males (63). This morphological change corresponds to a higher expression of pro-inflammatory markers in male microglia. When circulating sex hormones are reduced through orchi/ovariectomies differences in genetic profiles are partially maintained. Further, when female microglia are transplanted into the male brain, the transplanted microglia maintain their female pattern of gene expression. Finally, the transplanted female microglia conferred a more neuroprotective response to ischemic injury when compared to male microglia transplanted back into male mice (63). These experiments demonstrate that early exposure to sex hormones can induce a sexual phenotype that function independent of circulating sex hormones, suggesting an epigenetic pattern of gene expression that is established early in development and affects reactivity to the environment.

Differentiating between organizational and activational changes is challenging. For decades, neuroendocrinologists have acknowledged that many experimental and clinical observations do not fall within a simple, two-process theory (54). This classification is further confounded in the context of SCI where the pathophysiology of secondary injury is not fully understood. Nonetheless, below we consider the activational effects of sex to begin to structure the framework for understanding sex as a biological variable in neurotrauma.

#### Males and Females Have Differing Inflammatory Profiles After Neurotrauma

Parallel bodies of literature in TBI and stroke support a protective effect of being female. Therefore, it remains likely that a similar, albeit small, sex-dependent effect exists following SCI despite inconsistent pre-clinical results. To understand why this may be the case, investigations have focused on the immunomodulatory role of sex hormones in neurotrauma. Because more work has been performed investigating sex-dependent inflammatory responses in TBI, compared to SCI, findings from TBI literature will be used to extrapolate interactions that may exert influence in SCI. There are, however, important differences between inflammatory responses occurring following TBI and SCI which have been reviewed in detail elsewhere (64). Briefly, in response to identical experimental lesions, SCI induces a larger total inflammatory response acutely following injury that propagates a greater distance from the lesion site and comprises a higher proportion of infiltrating leukocytes and myeloid cells (65, 66). With consideration of these fundamental differences between SCI and TBI, a sex-dependent inflammatory response in neurotrauma is supported by increased inflammation in male mice acutely following TBI (50, 51). Specifically, in response to TBI, male mice exhibit a larger total inflammatory response arising from both microglia and myeloid cell infiltration with a proportionally larger increase in myeloid cells at 1-DPI and microglial proliferation at 3-DPI relative to females. This acute microglial-specific inflammatory response in males is concurrent with what would be expected given a suppressive role of estrogens on microglial activation (67, 68).

##### Estrogens have immunosuppressive properties

Estrogens and progesterone have been thoroughly investigated as neuroprotective agents due to their role in activating pro-survival pathways as well as by exerting anti-inflammatory and antioxidant properties directly (43, 69–71). Indeed, estrogens mitigate inflammation after SCI in part by interfering with inflammasomes (48). Similarly, estrogens exert both direct and indirect effects on mitochondrial function regulating cellular metabolism (72–74) which is becoming increasingly attributed to inflammatory activation and exacerbation of secondary injury following neurotrauma (75, 76).

Estradiol, a main estrogenic hormone in mammals, influences several immune cell types either directly or indirectly, including B cells, T cells, macrophages, NK cells, and eosinophils (77). Additionally, estradiol increases Th2 type cytokines and accordingly decreases cell-based immunity in both animal models and humans (78). This pattern of immune regulation suggests that estradiol decreases the expression of pro-inflammatory cytokines and reduces cell-mediated immunity and microglial activation. Cell culture studies have shown that physiological levels of 17β-estradiol *in vitro* significantly decrease microglial activation in response to immune stimuli (78). Recently, estradiol has also been shown to modulate neuroinflammation caused by TBI via the G protein-coupled estrogen receptor (GPER), which inhibits the expression of pro-inflammatory cytokines (IL-1β, IL-6, and TNF-α) and upregulates the anti-inflammatory cytokine, IL-4, consistent with a classically defined M2 phenotype (79). The role of estrogens as anti-inflammatory hormones does, however, contradict known clinical literature that suggests females mount a larger innate and adaptive immune response during infection and disease (80, 81). This paradox, in the context of neurotrauma, is not well-understood; however, the effects of estrogens on inflammation are postulated to be cell-type specific and dependent upon concurrent activating stimuli (82). For example, although estrogen receptors (ER) exist on both monocyte- and microglial-derived macrophages, estrogens suppress microglial activation through ERβ while, in contrast, activate monocytes through ERα (67, 68, 83, 84).

##### Estrogens modulate the adaptive immune response following SCI

T-cells, and other adaptive immune cells, may also exert sex-dependent effects on SCI recovery (16). In the absence of injury, females exhibit a different resting inflammatory profile consisting of higher CD4/CD8 T-cell ratios compared to males; however, overall, males have more total T-cells (85). Despite lower total T-cells at rest, females mount a stronger adaptive immune response, stimulating higher levels of T- and B-cell activity (81). This is evident in the higher prevalence of autoimmunity amongst females, with reports ranging from 60 to 90% of individuals with autoimmune conditions being female depending on the condition (86). SCI increases the likelihood of developing multiple sclerosis, an auto-immune condition, by 624% compared to the non-injury conditions, a frequency of 17.6-SCI vs. 2.82-non-injured in every 100,000 (87); however, whether being female increases this frequency risk has not been determined. Both B- and T-cells interact during the adaptive immune response after SCI with B-cells producing autoantibodies and T-cells reacting to myelin basic protein and other CNS proteins (88–92). Schwartz and colleagues argue that the adaptive immune responses to SCI are protective for females. This has been supported previously as functional differences between male and female rodents diminish upon experimental depletion of T-cells (16). Whether T-cells contribute toward recovery in females, or toward pathology in males, is not well-understood. However, Schwartz and colleagues report that injection of auto-activated T-cells against myelin-derived proteins improves functional and histopathological outcomes independent of sex (93).

##### Testosterone may exert sex-specific effects in SCI

Similar to estrogens and progesterone, testosterone also exerts immunomodulatory influence by suppressing monocyte-derived macrophages through downregulation of TLR-4 (94). The immunosuppressive activity of testosterone is suggested to contribute to more frequent bacterial infections and longer recovery periods from illness in males compared to females (95, 96). Low serum testosterone inversely correlates with the extent of circulating inflammatory cytokines (97), which pre-dispose men with low testosterone to an increased prevalence of metabolic syndrome (98, 99). Although it may be compelling to posit that an anti-inflammatory effect of testosterone can mediate protection against SCI, little evidence exists to support this hypothesis. In fact, although limited, publications investigating the influence of testosterone on functional outcomes after SCI support the modest immunosuppressive activities as detrimental to recovery (16). The inflammatory response to SCI facilitates both repair and exacerbates damage (100). Currently, not enough is known regarding how testosterone affects inflammation following SCI to conclude whether these effects mediate a net toxic or beneficial outcome.

Whether sex-differences in inflammatory profiles persist chronically after SCI remains undetermined. The data reviewed above indicating a male-dependent acute microglial proliferation following TBI, along with a strong link between microglia and developing neuropathic pain following SCI (101), merits further investigation to determine if similar sex-dependent inflammatory events translate into SCI. Due to the influence of sex hormones on inflammation, it may be necessary to tailor treatment strategies targeting inflammatory cascades to sex-dependent mechanisms.

#### Testosterone Mediates Sex Dependent Effects on SCI Recovery

In contrast to estrogens and progesterone, how androgens mediate sex-dependent effects in SCI is less studied. Whether testosterone exhibits an overall neuroprotective or detrimental effect on recovery from SCI remains controversial (43, 102). Current evidence supporting testosterone as potentially neurotoxic comes from the observation that castration of male rodents pre-SCI improves locomotor recovery, an effect that was further abrogated following exogenous delivery of dihydrotestosterone (16). Similarly, providing male rodents with an androgen receptor antagonist, Flutamide, significantly improves open-field motor scores when compared to placebo-treated controls, further suggesting a detrimental effect of testosterone on recovery from SCI (16). Importantly, this study replicated effects in both rats and mice, demonstrating a conservation of a biological process. Additional support for testosterone's potential toxicity has been found *in vitro*. Treating cultured oligodendrocytes with AMPA receptor agonists induces a mild excitotoxic response which is amplified when co-treated with testosterone (103). This may suggest that testosterone can sensitize white matter to the excitotoxicity that accrues following SCI. Indeed, testosterone has been demonstrated to exacerbate neurotoxic effects in other animal models of disease (104). Testosterone is affiliated with decreasing antioxidant responses via downregulation of Nrf2 in the presence of oxidative stress (104). This is in line with reports suggesting that age-induced decreases in the cellular antioxidant glutathione are significantly exacerbated in males compared to females (105). Indeed, glutathione levels are decreased in males compared to females in Alzheimers-like disease pathologies (106). Collectively these studies implicate testosterone as exerting net detrimental effects on SCI recovery, potentially through exacerbating secondary tissue damage (16, 102).

In contrast, some beneficial effects of testosterone have been found when analyzing systems away from the SCI lesion. SCI induces dendritic atrophy of lower motor neurons when de-innervated from supra-spinal connections (107). Adult female rats treated with testosterone abrogated this shortening of dendritic length in lower-motoneurons following SCI (47, 108). Similarly, exogenous testosterone administration following SCI protects against muscular atrophy (108) which can aid in recovery during periods of rehabilitation (109). Muscular support from dihydrotestosterone administration also improves bladder voiding capacity in rats, which may or may not be a desirable outcome in patient populations (47). Many of these outcomes support the role of testosterone as beneficial in chronic stages of SCI rather than as a beneficial mediator of acute injury and recovery. These contrasting outcomes reflect the complexity of the organizational and activational effects of sex hormones on SCI pathophysiology.

#### Response to Pharmacological Therapy After SCI Is Sex Dependent

##### Sex differences in cellular biology effect SCI treatment responses

Organizational differences between sexes, which arise from developmental exposure to sex hormones, elude to a probability that efforts to treat SCI may be sex dependent. This has been demonstrated in one experiment by treating SCI mice with pioglitazone, a diabetes drug otherwise used to enhance insulin sensitivity but also exerts analgesic effects (31, 35, 110). As mentioned above (section Pre-clinical Data Indicate that Sex-Differences are Outcome Specific), treating SCI with pioglitazone significantly attenuates pain in female mice without exerting a significant effect to male mice (31). This is consistent with similar sex-specific effects of pioglitazone exerting stronger insulin-sensitizing responses in females compared to males (35). Pioglitazone's biological target, peroxisome proliferator-activated receptor-γ (PPARγ), is known to interact with estrogen receptors in several ways. First, cytosolic ERα and ERβ bind and suppress PPARγ, interfering with its capacity to upregulate genes affecting adipogenicity (111–114). Next, downstream signaling of estradiol itself upregulates PPARγ (115), yet despite this interaction, levels of PPAR expression demonstrate sex-dichotomy in a tissue-dependent manner (116–118). The antagonistic nature of ER receptors to PPARγ's genetic influence may preclude a genetic mechanism as the underlying analgesic effects of pioglitazone. This has been supported by co-delivery of anisomycin with pioglitazone to stop new protein synthesis, which did not affect pioglitazone's analgesic effects in a mouse model of peripheral nerve injury-induced pain (110). This suggests that mechanisms underlying pioglitazone's analgesic effects are not acting through genomic influence and that either activated PPARγ has non-genomic effects or that pioglitazone acts on other unidentified targets in a sex-dependent manner. Although several other non-PPARγ targets have been identified for pioglitazone or other thiazolidinediones (TZDs) (119, 120), blocking PPARγ with a specific antagonist, GW9662, does mitigate analgesic effects derived from pioglitazone (110) confirming a PPARγ dependent mechanism of analgesia that is not transcriptionally dependent. Several cytosolic protein kinases have been shown to activate upon administration of TZDs, which can exert a wide influence of pioglitazone on cellular functions that may or may not be PPARγ dependent (119, 120). Why and how TZDs exert a sex-dependent effect remains unknown, but differential expression of any targets activated by TZDs may underly the sex-dichotomous effects that are observed both in clinical patients treated for diabetes or in animal models of SCI and pain. Taken together, sex-dependent effects of pioglitazone can serve as one example of how biological differences between females and males interact to affect treatment outcomes.

##### Sex differences in drug metabolism affect pharmacodynamics

Regardless of how sex hormones may influence drug effects, systemic differences between males and females exist that influence pharmacodynamics. Sex-based differences in absorption, metabolism, sequestration by plasma proteins, and clearance all interact to determine the availability of drugs on their intended targets post-administration (53). Although some differences in pharmacodynamic processing may be attributed to weight alone, standardizing drug delivery by bodyweight does not account for all disparities between males and females. Specifically, body composition plays an important influence, as on average women present with higher body fat percentages which can interact to affect a drug's pharmacokinetic profile (121). Differences in drug metabolism between males and females can be so large that these innate differences have been attributed to females experiencing higher frequencies of overdose and adverse events following drug delivery (122). Implications for these potential innate differences in drug metabolism extend into pre-clinical study design. Specifically, when both sexes are included in drug-delivery research, it is important to consider that optimal doses may differ, and if separate dose-dependent responses were not investigated, to consider how differences in drug metabolism may affect results. Differentiating between how cellular mechanisms and pharmacological dynamics affect sex-dependent responses to drugs will be difficult to elucidate but should be kept in consideration with study design and interpretation.

#### Sex Dependent Effects of SCI Change With Age

**Figure 2:**
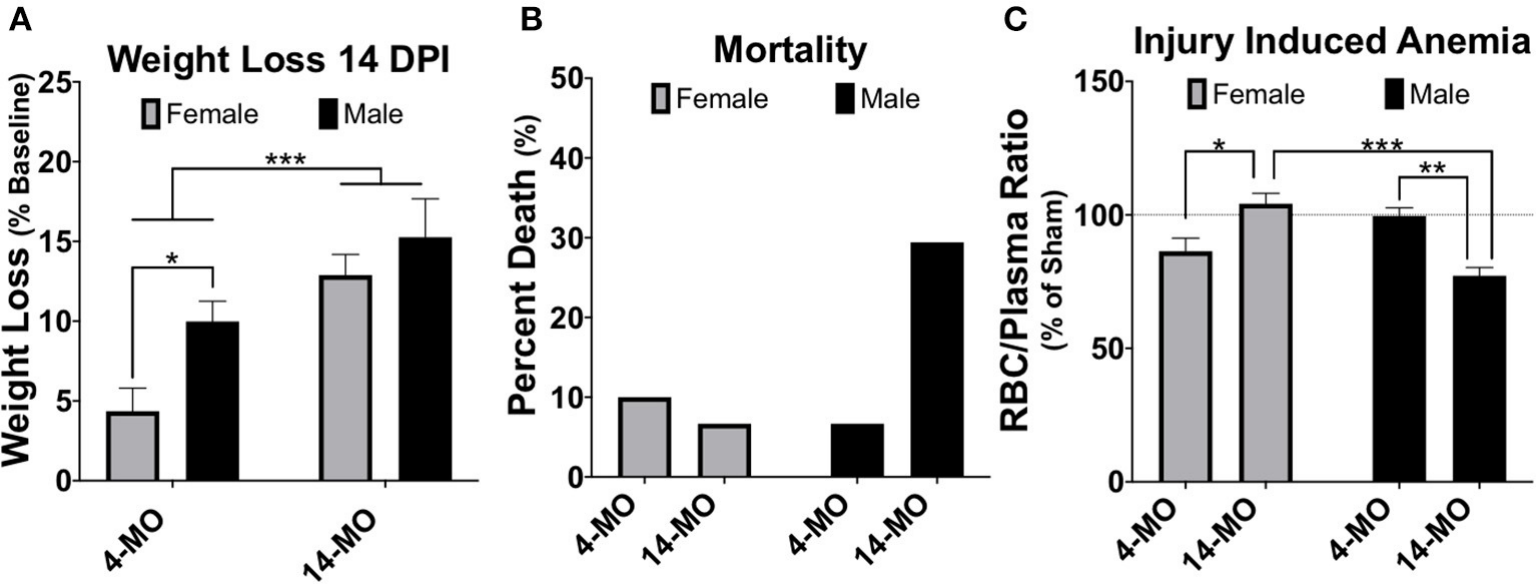
Weight loss, mortality, and injury induced anemia were greatest in 14-MO C57Bl/6J mice after 60 kDyn spinal contusion. Data was accumulated over two studies evaluating how age and sex affect outcomes after SCI. Mortality among mice were counted if found dead in cage or reached moribund euthanasia criterion. At 28-days post-injury (DPI) blood was extracted from the right atria via cardiac puncture and collected in EDTA tubes prior to trans-cardial perfusion. Red blood cells (RBC) were pelleted and the volumetric ratio of RBC to plasma was measured. Analyses were performed using two-way ANOVA with Tukey's *post-hoc* comparison. **(A)** Main effects of age (*p* < 0.001) and between 4-MO female- and male-mice (*p* < 0.05) suggest that being an older male facilitates the greatest weight loss after SCI (*n* = 9–10). **(B)** 14-MO males experience ~ 30% mortality (*n* = 5/17) after SCI, predominately within the first week after injury, where-as 4-MO male- (6.66%; *n* = 1/15) and female- (10.0%; *n* = 2/20), as well as 14-MO female-mice (6.66%; *n* = 1/15) experience less SCI-induced mortality. **(C)** After normalizing RBC ratios to same age sham-controls (*n* = 5) 14-MO-male mice experienced the greatest injury induced decrease in RBC/Plasma ratios with a significant sex-by-age interaction demonstrating a sex-divergent response to aging after SCI [(*n* = 9–10); *p* < 0.001]. Mean ± SEM. *p < 0.05; **p < 0.01, ***p < 0.001.

The combined protective effects of estrogens with potentially toxic effects of testosterone have important implications for how additional organizational changes with age may influence sexual dimorphisms to SCI. Net effects of decreased estrogens and testosterone with age could reciprocally influence recovery, however this remains to be determined. Increased age at the time of injury is known to impair functional recovery following SCI in female rodents (10–12, 123–128), however, no pre-clinical work has been done to evaluate if aging changes or exacerbates sex-dependent differences after SCI. Current ongoing projects in our lab are seeking to address this literature gap and have compiled mortality and weight loss data from several ongoing studies. We find that older age increases SCI-induced mortality in males and but normalizes sex differences in weight loss found at younger ages (Figures 2A,B). Specifically, we found significant main effects of age [*F*_1, 34_ = 17.61; *p* < 0.001] and sex [*F*_1, 34_ = 5.89; *p* < 0.05) for weight loss at 14-days post-injury (DPI), with 4-MO males losing significantly more weight compared to 4-MO females (*p* < 0.05) and no sex differences in 14-MO mice (*p* < 0.53). Previous meta-analyses of clinical data have supported this increased mortality amongst men post-SCI (6), with age serving as a strong predictor of early mortality (129).

Mechanisms underlying a sex-dependent increase in mortality remain unknown. We postulate that this may be in part due to an undetermined interaction between decreased testosterone and age. Both aging and SCI are known to decrease free testosterone (130–132). Whether or not there is a compounding decrease in testosterone following SCI in older males has yet to be determined. This decrease in testosterone with age and injury has important implications on the maintenance and regulation of erythropoiesis (133, 134). Indeed, aged male C57BL/6 mice have been proposed to be used as a model of anemia due to this reduced testosterone-erythropoiesis interaction (135). We have found supporting evidence that SCI induces a concurrent reduction in crude red blood cell/plasma ratios (RBC/plasma) in aged male mice at 28-days following SCI (Figure 2C). Specifically, we found a significant sex by age interaction [*F*_1, 33_ = 27.61; *p* < 0.0001] with 14-MO mice increasing RBC/plasma ratios compared to 4-MO mice in females (*p* < 0.01), but decreasing in males (*p* < 0.01). This fits our empirical observations that older male mice appeared colder to the touch during routine bladder care for a few days following SCI compared to other groups, during which time an increase in mortality was observed. A more thorough investigation is required to follow up on these early findings. Overall, the reciprocal role of how SCI affects hormone balance and implications on acute and long-term management of paralysis has not been well-investigated, and even less has been done to determine how this might compound with age.

### SCI Alters Sex Hormones After Injury

#### SCI Induces Estrous Cycle Dysfunction and Reduces Estradiol

In addition to understanding how sex hormones may affect SCI outcomes, it is also consequential to consider activational changes in sex hormones after SCI. Because circulating levels of sex hormones regulate a breadth of health outcomes ranging from depression to inflammation and osteoporosis, it is important to better understand how SCI might mediate acute or chronic perturbations to hormonal regulation. While the largest regulator of sex hormones arises from coordinated paracrine activity of the hypothalamo-hypophysial system, neural innervation of the gonads co-exists as a modest contributor (136). This leads to multiple possible mechanisms arising after SCI which can affect both acute and chronic hormonal regulation. First, systemic inflammation and stress experienced acutely following SCI elevate glucocorticoids which regulates estrogens and progesterone production by decreasing the sensitivity of ovaries to luteinizing hormone and decreases the effectiveness of aromatase (137–139). Next, direct de-innervation from brainstem nuclei can permanently abolish supraspinal control over hormone regulation. Whether changes to the hypothalamo-hypophysial system are maintained chronically post-SCI is not well-investigated, however, atrophy of the gonads (hypogonadism) is frequently reported in men following SCI, while effects of SCI on ovaries remain unreported in humans. Two studies report the effects of chronic SCI on rat ovarian tissue and found an overall decrease in volume, corresponding to a decreased diameter of the follicle, ovum, and thickness of granulosa layers, with a concurrent increase in follicular atresia (140, 141).

**Figure 3:**
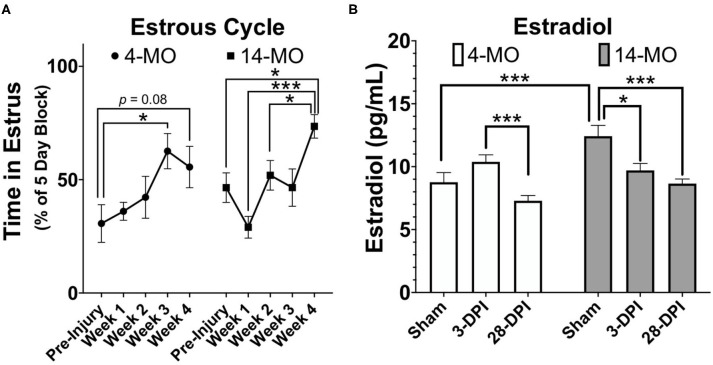
SCI (60 kDyn contusion) induces estrus cycle dysfunction **(A)** concurrent with decreased plasma estradiol by 28-DPI **(B)** in C57Bl/6J female mice. **(A)** Estrus cycle monitoring was performed for 28-DPI throughout the week and analyzed for estrous stage as previously described (144). Percent of time spent in estrous throughout a consistent 5-day block each week was assessed and used for analysis. Two-way repeated-measures ANOVA was used for analyses and revealed a significant main effect of time (*p* < 0.001) with both 4- and 14-MO mice demonstrating a significant increase in time spent in estrous compared to pre-injury conditions (*p* < 0.05; *n* = 9–10). Mean ± SEM. *p < 0.05; **p < 0.01, ***p < 0.001.

SCI dysregulates estrous cycling in rats, resulting in prolonged cycle duration (142, 143). By blocking time into week intervals post-SCI, we have found similar results in mice that SCI expands time spent in the estrous phase of the cycle [*F*_4, 36_ = 6.74, *p* < 0.001; Figure 3A] with a significant increase found by 28-DPI (*p* < 0.001) compared to pre-injury levels when age is combined. When comparing within an age, 4-MO mice reached a significant increase in time spent in the estrous phase compared to pre-injury levels by 21-DPI (*p* < 0.05) and 14-MO mice reached significance by 28-DPI (*p* < 0.05). Correspondingly, we also found a time by age interaction [*F*_2, 33_ = 6.08, *p* < 0.01; Figure 3B] in the plasma estradiol response to SCI likely owing to a modest increase in estradiol in 4-MO-, but a significant decrease in 14-MO female mice at 3-DPI (*p* < 0.05). Only 14-MO mice had a significant decrease in plasma estradiol levels at 28 days post-SCI compared to pre-injury values (*p* < 0.001). An inverse relationship between increased cycle duration and decreased estradiol is compatible with hormonal feedback mechanisms. Estrogens increases during pro-estrus until critical concentrations trigger an LH surge and ovulation, facilitating a transition into estrus. Therefore, decreased plasma estradiol will result in prolonged cycle duration which may delay the onset of an LH surge (145–147).

Decreased estrogens post-SCI may exert chronic influences over maintaining bone mass, as well as regulating metabolism and weight. First, reduced estrogens decreases bone density and is strongly associated with developing osteoporosis in postmenopausal women (148). After SCI, decreased bone density and osteoporosis are known consequences of reduced-weight bearing, however, women lose significantly more bone density compared to men by 5-years post-injury (52). Whether SCI-induced decreases in estrogens underlie these sex-based differences has not been determined. Decreased estrogens may also play a role in slowed metabolism that occurs following SCI (72, 149). Although a slowed metabolic rate is consequential to decreased physical activity, muscular atrophy, and limited weight-bearing after paralysis, a decrease in estrogens after SCI may exert a significant contribution to metabolic dysfunction and chronic health complications in women.

#### SCI Decreases Testosterone

Similar to female sex hormones, testosterone levels in males decrease acutely following SCI (150) and maintain at lower levels in some men throughout a lifetime (130, 131). However, the levels of testosterone measured among men with SCI is varied across studies. Most reports suggest men with SCI have significantly lower testosterone levels than uninjured counterparts (130, 131, 151) with testosterone levels being lower in motor complete compared to incomplete individuals (151). In contrast, older reports found no differences in testosterone levels after SCI (152, 153). Indeed, Kikuchi et al. (152) found that all but one male SCI patient (*n* = 15) were within normal range when compared with age-matched controls. A possible explanation for this discrepancy comes from evidence showing that testosterone is lowest acutely after SCI and gradually increases over 18 months' time (154). A recent review found the prevalence of men with low testosterone acutely after SCI ranged from 69 to 83% of patients, which contrasts a prevalence of 10–46% of men with chronic SCI (155). Why testosterone decreases acutely following SCI and/or maintains at low levels has yet to be elucidated. However, elevated levels of corticosterone/cortisol may drive a decrease in testosterone acutely following injury (156), while chronic decreases in testosterone may arise from physical inactivity and muscular atrophy.

Implications for an acute decrease in testosterone following SCI are not well-defined, however chronic decreases in testosterone can result in increased visceral adipose tissue, metabolic syndrome, and depression (130, 155). Men with SCI and low serum testosterone (defined as <400 ng/dL) have higher total body fat percentage, particularly in the trunk area (9.6 and 12.7% higher compared to normal range testosterone, respectively), while men with SCI and normal serum testosterone levels have decreased muscle atrophy (108), increased motor function (157) and a better cardiometabolic profile when compared to men with SCI and low testosterone (158). Whether these findings are more indicative of the extent of physical activity and rehabilitative training after SCI is not clear.

### Incorporating Both Sexes in SCI Research: Experiences and Recommendations

#### Role of Monitoring and Manipulating Sex Hormones

As basic and pre-clinical neurotrauma research data accumulates on both males and females, there will likely be a surge of unexpected sex-dependent interactions that will help guide efforts to develop personalized medicine. When a research question is not aimed at understanding sex-based differences it may be not be feasible to incorporate thorough analyses beyond just including both sexes. Manipulation of sex hormones through orchi/ovariectomies, pseudopregnancies, or injection of male/female hormones need not be included in studies that do not have a central hypothesis about understanding sex-dependent effects. However, simple additions to data collection can help gather vital information regarding how sex hormones affect outcomes of biological phenomena and treatment, even if significant effects are underpowered in any given study. The field of SCI has established an open data commons for depositing information from research studies which is being used to mine big-data sets gathered across multiple neurotrauma centers [(159, 160), ODC-SCI^1^]. The more data which enters these public domains, the higher probability exists to derive meaningful relationships that may have not been directly evident within the scope of a given study. For example, mining of clinical data of patients with SCI revealed a significant relationship between mean arterial blood pressure and functional outcomes; this has yielded re-consideration of clinical guidelines for maintaining blood pressure acutely after SCI (161).

Regarding collecting data and monitoring of sex hormones and/or estrous cycles, there are simple ways to incorporate these elements into a research design without substantial increases in time or cost. A simple method to determine the stage of the estrous cycle is through vaginal lavage and visual analysis of cellular morphology, which takes only seconds to perform per animal (144). As mentioned above, drugs can interact with sex-hormone signaling in robust ways, therefore determining the state of estrous at time of SCI and/or intervention can aid in better predicting how estrous cycles can affect therapeutic efficacy. To measure specific hormone levels, small volumes of accumulated plasma can be used to determine concentrations of sex-hormones using commercially available ELISA kits or services available within university/hospital departments, or available at other institutions for small fee's (e.g., see University of Virginia's Center for Research in Reproduction Ligand Assay and Analysis Core). Data derived from such efforts in collection and reporting may quickly accelerate our understanding of how biological diversity can affect outcomes to injury and treatment.

#### Age or Weight Matching in Analysis

Male rodents are larger than female rodents by nature. This creates complications for interpreting sex-based studies in a number of ways and leaves unresolved questions about whether it is most appropriate to age or weight match. While aging mice can appropriately equalize weights between groups, aging is known to negatively affect many biological processes that will interfere with recovery and is therefore not a recommended strategy for comparison. However, heavier animals often mean larger spinal cords. Different size spinal cords between groups may affect injury dynamics and leave questions regarding how increased muscle mass or weight may affect recovery potential. One study has evaluated how different sizes of spinal cords affect injury dynamics and suggested that larger spinal cords arising from increased age at time of injury did not affect displacement of the cord during injury (162). Because a similar displacement of a larger cord would mean that a smaller percent is being displaced, these findings leave ambiguity regarding how injury dynamics are affected by spinal cord size. Larger cords can also introduce systematic bias when analyzing histopathology's that need to be considered. For example, if using total amount of spared tissue surrounding the lesion epicenter as an outcome, it remains possible for a larger cord to have the same total area of spared tissue as a smaller cord, but less percentage of spared tissue based on original volume (162).

Whereas, it may appear appropriate to standardize obtained area values to the percent of the total section, this may not be possible or advisable for several reasons. First, if performing work in animal models of SCI that form cystic cavitation, the extent of atrophy and cavitation surrounding the lesion epicenter will interfere with deriving an accurate percentage of spared tissue and can be unintentionally manipulated during staining. This is a complication previously discussed when comparing rats with different size cords as a confound of age (162). If performing SCI work in a mouse, which forms fibrotic lesion cores, the extent of inflammation and swelling within the lesion can interfere with deriving an accurate percentage. It may be possible for the lesion core to expand while maintaining a consistent spared tissue volume, resulting in a perceived lower percent of spared tissue if standardized to the total area of that particular section. For these reasons and others, when comparing between sexes it is best to obtain tissue from uninjured areas of the cord or ideally from sex-matched naïve/sham-injured mice for normalization and to control for unpredictable error that can confound interpretation.

#### Housing Considerations and Effects of Single vs. Group Housing on Outcomes

**Figure 4:**
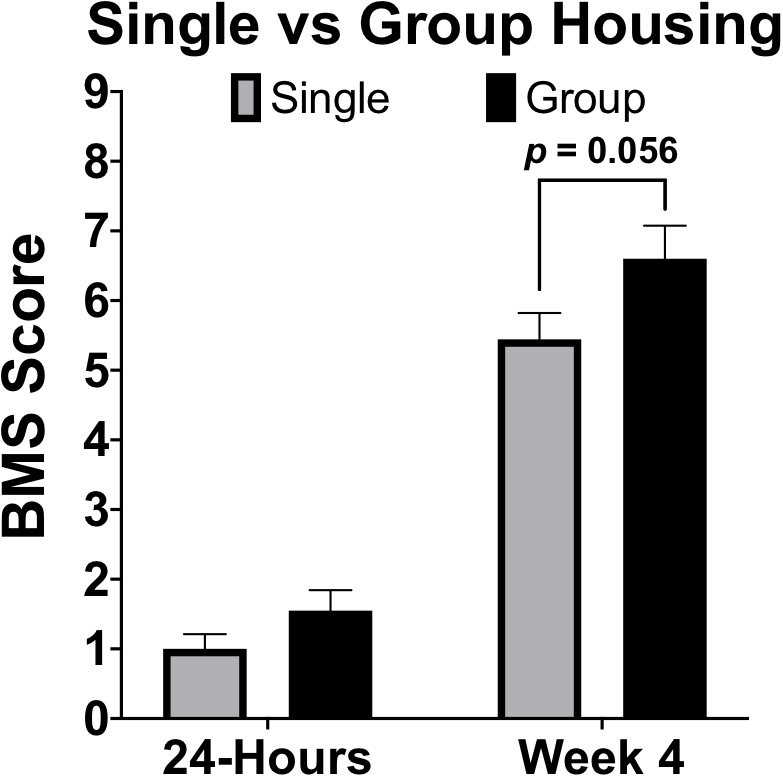
Single housing of female C57Bl/6J mice receiving moderate (60 kDyn) contusive SCI recovered less motor functions compared to group housed mice by 28-DPI. Data was compiled from two independent studies utilizing the Basso Mouse Scale [BMS; (165)] as an assessment of locomotor recovery in single and group housed conditions. Although differences were marginal (*p* = 0.056; *n* = 9–10), this data demonstrates how single housing may affect motor recovery and emphasizes the importance of housing all groups comparably if between group comparisons will be made. Mean ± SEM.

Housing conditions in rodent models of SCI can exert large influences on recovery. Effects of environmental enrichment have demonstrated that single housing significantly decreases SCI recovery relative to group and environmentally enriched conditions (163). In many animal models it is common to single-house males due to aggression, or limit group housing of males due to size restrictions in the home cage (164). When comparing between sexes it is therefore necessary to consider how animals will be housed and to ensure a standard of housing for all sexes. Most importantly, if animals will be group-housed, determine *apriori* how aggressive conflicts will be resolved in a manner that does not result in isolation of a large proportion of one sex. In most published rodent studies that have evaluated for sex differences after SCI, no statements were made regarding if both sexes were housed comparably. We have compiled locomotor data at 28-DPI from two independent studies, one with group-housed females (4–5 mice/cage), the other with single-housed mice. Although marginal, differences in locomotor function [BMS scores; (165)] at 28-DPI were smaller in single-housed mice (*p* = 0.056; Figure 4). To ensure that observed sex-based effects are not actually a manifestation of differences in housing conditions, it is imperative to treat all animals of both sexes the same and not overlook small details such as housing conditions.

#### Statistical Concerns and Study Design

Several design strategies exist to account for adding sex as a biological variable, however, the best approach is often the most rigorous: performing multi-factorial design such as two-way ANOVA instead of combining sexes or presenting one-way ANOVAs by group. Consequently, especially if the study is powered for a sex effect, this may require increased sample sizes to compensate for a loss of power, demand more financially, and require more time to complete a study. Because this does increase design complexity, there are emerging demands unique to both authors and readers for appropriate interpretation of study results.

A strategy often used to circumvent more complicated multi-factorial statistical methods is to test for the existence of a biological phenomenon or treatment effect by limiting comparisons within a single sex and running analyses in males and females in parallel. Whereas, this strategy may sufficiently reduce a need for more complicated multi-factorial statistics, conclusions derived from this study design are limited and do not allow for an accurate comparison of between-group effects, nor does it allow for detecting meaningful interactions (166, 167). Indeed, established journals are increasingly considering such statistical strategies as erroneous and are asking for direct comparisons to be made between groups if conclusions will be drawn about between-group effects (168). In other words, it is becoming increasingly unacceptable to make a claim that “*drug A exerted a significant effect in group X, but not group Y*” as a statement to suggest that a drug was only or more effective in one group. This criticism holds merit and can be understood using an extreme hypothetical situation. Assuming males have less variability in outcomes compared to females, a neuroprotective drug could improve motor outcomes consistently by 10% in males but inconsistently by 30% in females, resulting in a significant improvement in males only. Where-as this may make a statement about the reliability of a drug exerting an effect, no consideration to effect size was given, and likely the increased variability in females could dictate that the study was merely underpowered. Similarly, this statistical strategy leads to an ease of misinterpreting *P*-values as a magnitude of effect. Where-as using a two-way ANOVA in this hypothetical situation may result in similar within-group effects, data would also be gained regarding an interaction or magnitude of effects between sexes which could better articulate that females responded more robustly to the drug. If single within-group comparisons are to be used, it remains important for authors to disseminate data in a manner appropriate for the dataset, and properly articulate if one group was underpowered by providing outcomes of effect size, variance, and observed power.

**Figure 5:**
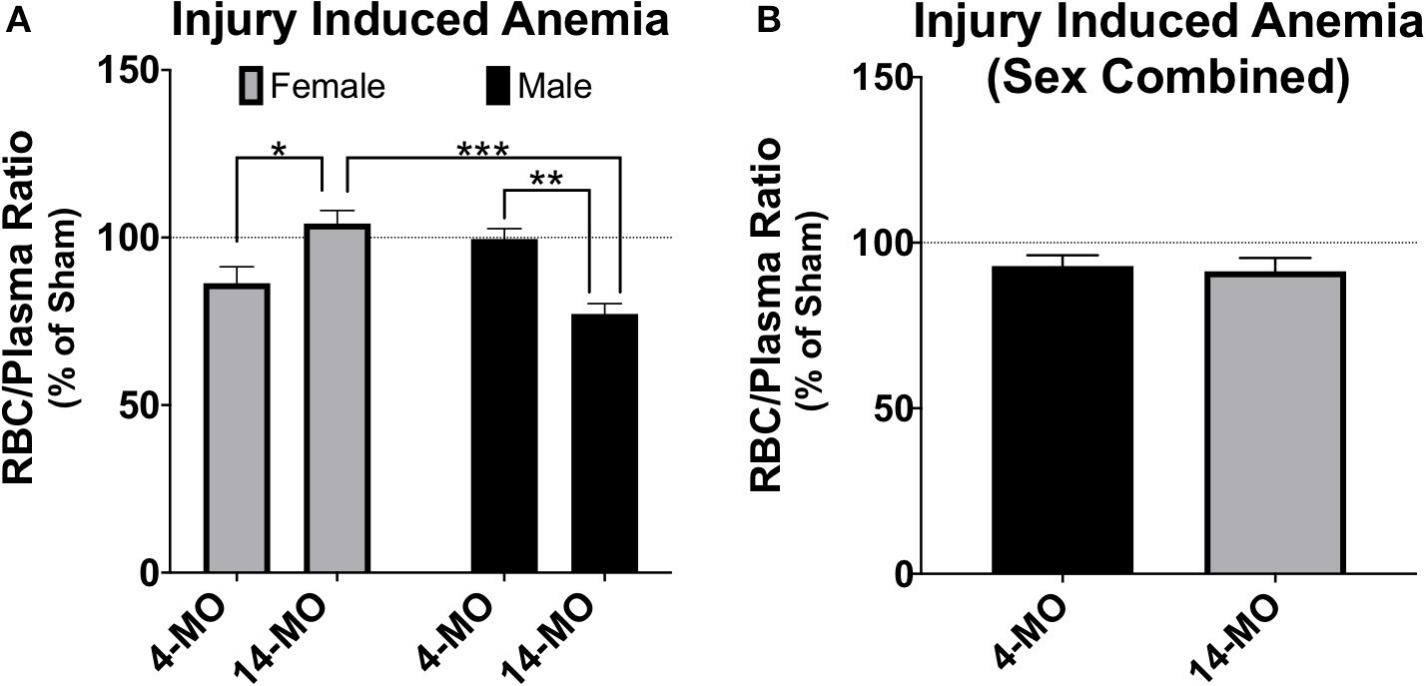
Analysis of RBC/plasma ratios using two-way ANOVA **(A)** demonstrates a significant sex-by-age interaction (*p* < 0.0001). When males and females are combined **(B)**, no effects are found either alone (*p* = 0.76), or when using sex as a categorical covariate (*p* = 0.73). While more power can often be gained by combining groups, sex-dependent interactions can mask main effects even when sex does not significantly explain variance in the model. This exemplifies problems that can emerge when combining sex to test a main hypothesis and argues for using factorial design strategies as a first approach to statistical analysis. Mean ± SEM. Tukey's *post-hoc* used for pairwise comparisons. *p < 0.05; **p < 0.01, ***p < 0.001.

A second commonly employed method is to combine sexes and/or use sex as a categorical covariate in analysis. Using sex as a covariate can determine whether sex is a significant predictor of variability in outcomes and perform an adjustment of mean values based on variability explained by sex. In some circumstances combining sexes can be a method to improve power, such as when little sex differences exist and is evidenced by sex not explaining much variability in a model. However, even when sex is not a significant contributor of variance, combining sexes can be problematic for several reasons. First, direct comparisons are not made between sexes. Next, adjusted means caused by both merging data and from covariate adjustments may wash out sex-dependent effects and lead to false conclusions that a response is either present or absent in both sexes. Here, again, the key idea is the inability for combined or covariate analysis to detect significant interactions. The pitfalls of merging data between sexes can be best articulated using an example from data provided in this review (Figures 2C, 5A). As this data is currently presented, a two-way ANOVA demonstrates a significant sex-by-age interaction (*p* < 0.0001), indicative of an increasing RBC/plasma ratio with age in females, but decreasing in males. If data from sexes were combined to only test for the effects of age, then the resulting *T*-test would not detect a difference between 4- and 14-MO mice (*p* = 0.76; Figure 5B). Similarly, when utilizing sex as a categorical co-variate to adjust mean values, the resulting ANCOVA would also not detect an effect of age (*p* = 0.73). The concepts emphasized in this example can be applied to situations where even moderate trends toward an interaction may obscure sex-dependent effects if data were to be combined.

In the example provided above, the interaction between the independent variables, age and sex, exert a reciprocal influence on the dependent variable, RBC/plasma ratio. In this case, combining sexes masked all effects in the model, which would lead to the false interpretation that neither age nor sex effect the RBC/plasma ratio after SCI. However, when combining sexes, it may also be possible for the reciprocal response to be true. Specifically, the magnitude of an effect from a single sex could carry a significant main outcome effect in the model, falsely indicating that the dependent variable increases in both sexes equally. In both of these conditions, when combining sexes, little to no information can be appropriately gained on sex effects, which can either eliminate detecting a main effect or mislead to suggest that an effect is ubiquitous between sexes. It is important to note that while this information can be obtained by utilizing multi-factorial approaches, often there may actually be no sex-dependent effects, whereby combining sexes can lead to a beneficial increase in power. However, in cases where sexes are combined, reporting the mean value and measurements of variance in each sex will help readers better understand sex-dependent relationships that may exist but were underpowered for detection in the study.

While multi-factorial approaches such as two-way ANOVA are recommended to test for both a sex effect and for possible interactions with the other independent variable, there are complications that may arise from additional pairwise comparisons. First, main effects in ANOVA can detect sex effects with greater power compared to individual comparisons. Next, if the method of *post-hoc* is not chosen carefully, irrelevant comparisons may be made resulting in further loss of power. Again, data provided in this manuscript (Figure 4) can be used to exemplify these points. Although this data was not comparing between sexes, our conclusion of a marginal group effect (section Sex Dependent Effects of SCI Change with Age, Figure 4, *p* = 0.056) is based on two-way ANOVA followed by pairwise comparisons using a Sidak correction for multiple comparisons. While technically correct, this use of pairwise comparisons is misleading and suggests that no differences between groups exist. From the two-way ANOVA model below (Table 1), we see that the statistically correct conclusion is that there is a significant group effect after adjusting for a strong time effect (*p* < 0.05). More specifically, being group housed, instead of single housed, increases the mean response by 0.42 (0.066, 0.78). Note that the confidence interval does not include zero. Further, in this particular example, time post-injury provides little value for individual pairwise comparisons within a group, because a significant recovery after SCI is common knowledge in the field. Similarly, comparing 4-week single housed to 24-h group housed, or vice-versa, provides no useful information. This can result in making several irrelevant comparisons if all groups are analyzed to each other during *post hoc* analysis, which results in a significant loss of power. Because this relationship between a loss of power from irrelevant comparisons can be amplified in more complicated studies, *post hoc* comparisons must be carefully selected. When sex is not the main hypothesis in a study, but is nevertheless included in analysis, it may often be may be best to limit pairwise comparisons within a sex and leave main and/or interaction ANOVA effects to determine between sex-effects.

**Table 1:** Two-Way ANOVA Model Single vs. Group Housing.

**Term**	**Estimate**	**95% Confidence Interval**	***P*-value**
Intercept	3.65	3.30, 4.01	<0.0001
Housing condition [Group vs. Single]	0.42	0.066, 0.78	0.0215
Label [24-h]	−2.38	−2.73, −2.02	<0.0001

Considering limitations to statistical strategies described above, it is still recommended to use multi-factorial approaches as a first pass to analyze data that includes sex as a biological variable. This will require more careful *a priori* power analyses which may be best approached by estimating animal requirements using groups and comparisons with the highest expected variability. When studies are not focused on detecting differences between sexes, power analyses should focus on finding within-group comparisons and powering studies accordingly to still use multi-factorial analysis. It may not be necessary to always power studies for between-sex effects, especially considering such small sex differences being reported in some outcomes after SCI. Although not encouraged, if data from both sexes must be combined, it should still be reported separately. Performing multi-factorial analyses will be the best methods moving forward to detect potentially meaningful sex-dependent effects.

With a more complicated statistical design conferred from including sex as a biological variable, both authors and readers assume a greater responsibility to appropriately interpret results. Including sex as a biological variable will complicate statistics and may result in studies that are insufficiently powered to detect a sex effect. An assumption that insignificant *P*-values mean no sex effects are present should not be inferred without excluding the possibility that the study is simply underpowered. This should be kept in consideration for interpreting both between and within sex effects. The NIH initiative to include sex as a biological variable will indubitably result in an emergence of studies that are underpowered to detect a sex effect. It is therefore important to interpret data with caution as to not ignore sex effects that may exist but were underpowered, or worse, erroneously interpret an effect as only present in a single sex if trends in the other sex suggest a mere lack of power. Finally, even when sound statistical measures are performed, if no differences between sexes are found, it is important to consider that biological mechanisms underlying those net effects may significantly differ, as described above, but were not revealed within the scope of the study.

## Summary and Concluding Remarks

In summary, both clinical and pre-clinical reports find that females recover more locomotor abilities after SCI. Much of this sex-dependent recovery has been attributed to the role of sex hormones on both neuroprotection and immune modulation. However, because inflammation mediates several modalities of SCI-induced dysfunction, there is an increased need to expand sex-based investigations into outcomes of pain, bowel, or bladder dysfunctions. Sex-differences in acute inflammation have been reported following TBI and similar effects are likely to be found following SCI. It remains to be determined if sex differences in acute inflammation are causal to a greater frequency of SCI-induced pain that is reported in females. Treating neuropathic pain arising post-SCI, however, can be sex dependent. A sex-dependency in treating SCI-induced pain with pioglitazone raises important concerns regarding the lack of inclusion of both sexes in pre-clinical SCI research. This is particularly concerning due to the incongruence between a male-dominated clinical base, and a female-dominated pre-clinical base. Inclusion of both males and females in pre-clinical SCI research is, therefore, essential to improve the translatability and predictability of treatment effects.

The contribution of sex hormones to the injury response has been the primary area of investigation when considering sex as a biological variable. However, SCI has also been reported to chronically reduce the circulation of sex hormones, which may have long term health consequences. How sex hormones effect injury, recovery, and the long-term health after SCI are mediated by differences between the actions of androgens and estrogens. The influence of sex hormones on neural development *in utero*, and throughout a lifetime, leaves both an organizational and activational footprint in the nervous system that may be important to better understand the sex-dependency of injury and intervention. Further, with advancing age comes a decrease in sex hormones that may exert unique sex-dependent considerations to injury, recovery, and health after SCI.

Ultimately, our ability to consider sex as a biological variable in the study of SCI will depend upon open and rigorous data reporting and interpretation. There are several technical confounds that should be considered in a study design including differences in anatomy, behavior, housing, and drug metabolism. Similarly, there are practical concerns regarding the appropriate statistical analysis for including sex as a biological variable that need to be accounted for, lest an inappropriate rejection of sex-dependent effects may be made, or important interactions may be missed. We have argued that sex should be included as a factor in SCI experiments and reporting should include results from multi-factorial analysis including interactions. As a field we must remain sensitive to the possibilities that underlying biological mechanisms of dysfunction can deviate substantially despite minimal differences in observable functional outcomes. Collective efforts to understand how sex affects SCI pathophysiology are emerging as new and exciting frontiers in neurology.

## Data Availability Statement

Data underlying findings from these studies will be made publicly available through the spinal cord injury open data commons after acceptance, as well as, by request (https://scicrunch.org/odc-sci).

## Ethics Statement

The animal studies were reviewed and approved by the University of Kentucky's Institutional Animal Care and Use Committee.

## Author Contributions

ANS, SM, AJS, JW, JG, and MW contributed to writing, editing, and literature review. ANS, SM, JW, and WB performed experiments and obtained data included in the manuscript. JG and MW obtained Funding. All authors: contributed to the article and approved the submitted version.

## Conflict of Interest

The authors declare that the research was conducted in the absence of any commercial or financial relationships that could be construed as a potential conflict of interest.

## Footnotes

^1^ODC-SC. *Open Data Commons for Spinal Cord Injury* [WWW Document]. scicrunch.org. Available online at: https://scicrunch.org/odc-sci (accessed April 9, 2020).

## References

[1] NIH NOT-OD-15-102.html (2015). grants.nih.gov. Available online at: https://grants.nih.gov/grants/guide/notice-files/NOT-OD-15-102.html (accessed April 12, 2020).

[2] Späni CB Braun DJ Van Eldik LJ. Sex-related responses after traumatic brain injury: Considerations for preclinical modeling. Front Neuroendocrinol. (2018) 50:52–66. 10.1016/j.yfrne.2018.03.006 29753798 PMC6139061

[3] Haberman S Capildeo R Rose FC. Sex differences in the incidence of cerebrovascular disease. J Epidemiol Community Health. (1981) 35:45–50. 7264532 10.1136/jech.35.1.45 PMC1052119

[4] Bracken MB Shepard MJ Collins WF Holford TR Young W Baskin DS A randomized, controlled trial of methylprednisolone or naloxone in the treatment of acute spinal-cord injury. Results of the second national acute spinal cord injury study. N Engl J Med. (1990) 322:1405–11. 10.1056/NEJM199005173222001 2278545

[5] Roof RL Hall ED. Gender differences in acute CNS trauma and stroke: neuroprotective effects of estrogen and progesterone. J Neurotrauma. (2000) 17:367–88. 10.1089/neu.2000.17.367 10833057

[6] Wilson JR Cadotte DW Fehlings MG. Clinical predictors of neurological outcome, functional status, and survival after traumatic spinal cord injury: a systematic review. J Neurosurg Spine. (2012) 17:11–26. 10.3171/2012.4.AOSPINE1245 22985366

[7] Chen Y He Y DeVivo MJ. Changing demographics and injury profile of new traumatic spinal cord injuries in the United States, 1972-2014. Arch Phys Med Rehabili. (2016) 97:1610–9. 10.1016/j.apmr.2016.03.017 27109331

[8] Furlan JC Krassioukov AV Fehlings MG. The effects of gender on clinical and neurological outcomes after acute cervical spinal cord injury. J Neurotrauma. (2005) 22:368–81. 10.1089/neu.2005.22.368 15785232

[9] Sipski ML Jackson AB Gómez-Marín O Estores I Stein A. Effects of gender on neurologic and functional recovery after spinal cord injury. Arch Phys Med Rehabili. (2004) 85:1826–36. 10.1016/j.apmr.2004.04.031 15520978

[10] Gwak YS Hains BC Johnson KM Hulsebosch CE. Effect of age at time of spinal cord injury on behavioral outcomes in rat. J Neurotrauma. (2004) 21:983–93. 10.1089/0897715041650999 15318998

[11] Zhang B Bailey WM McVicar AL Gensel JC. Age increases reactive oxygen species production in macrophages and potentiates oxidative damage after spinal cord injury. Neurobiol Aging. (2016) 47:157–67. 10.1016/j.neurobiolaging.2016.07.029 27596335 PMC5075497

[12] Zhang B Bailey WM McVicar AL Stewart AN Veldhorst AK Gensel JC. Reducing age-dependent monocyte-derived macrophage activation contributes to the therapeutic efficacy of NADPH oxidase inhibition in spinal cord injury. Brain Behav Immunity. (2018) 76:139–50. 10.1016/j.bbi.2018.11.013 30453022 PMC6348135

[13] NSCISC The 2019 Annual Statistical Report for the Spinal Cord Injury Model Systems. (2020). Available online at: https://www.nscisc.uab.edu/public/2019%20Annual%20Report%20-%20Complete%20Public%20Version.pdf (accessed March 31, 2020).

[14] Datto JP Bastidas JC Miller NL Shah AK Arheart KL Marcillo AE. Female rats demonstrate improved locomotor recovery and greater preservation of white and gray matter after traumatic spinal cord injury compared to males. J Neurotrauma. (2015) 32:1146–57. 10.1089/neu.2014.3702 25715192 PMC4507304

[15] Farooque M Suo Z Arnold PM Wulser MJ Chou C-T Vancura RW. Gender-related differences in recovery of locomotor function after spinal cord injury in mice. Spinal Cord. (2006) 44:182–7. 10.1038/sj.sc.3101816 16130019

[16] Hauben E Mizrahi T Agranov E Schwartz M. Sexual dimorphism in the spontaneous recovery from spinal cord injury: a gender gap in beneficial autoimmunity? Eur J Neurosci. (2002) 16:1731–40. 10.1046/j.1460-9568.2002.02241.x 12431226

[17] Datto JP Shah AK Bastidas JC Arheart KL Marcillo AE Use of the catwalk gait device to assess differences in locomotion between genders in rats inherently and following spinal cord injury. Dataset Pap Sci. (2016) 2016:6276348 10.1155/2016/6276348

[18] Emamhadi M Soltani B Babaei P Mashhadinezhad H Ghadarjani S. Influence of sexuality in functional recovery after spinal cord injury in rats. Arch Bone Jt Surg. (2016) 4:56–9. 26894220 PMC4733237

[19] Walker CL Fry CME Wang J Du X Zuzzio K Liu N-K. Functional and histological gender comparison of age-matched rats after moderate thoracic contusive spinal cord injury. J Neurotrauma. (2019) 36:1974–84. 10.1089/neu.2018.6233 30489213 PMC6599384

[20] Burke D Lennon O Fullen BM. Quality of life after spinal cord injury: the impact of pain. Eur J Pain. (2018) 22:1662–72. 10.1002/ejp.1248 29770520

[21] Hicken BL Putzke JD Richards JS. Bladder management and quality of life after spinal cord injury. Am J Phys Med Rehabil. (2001) 80:916–22. 10.1097/00002060-200112000-00008 11821674

[22] Cobo Cuenca AI Sampietro Crespo A Virseda Chamorro M Martín Espinosa N. Psychological impact and sexual dysfunction in men with and without spinal cord injury. J Sex Med. (2015) 12:436–44. 10.1111/jsm.12741 25388531

[23] Jain NB Sullivan M Kazis LE Tun CG Garshick E. Factors associated with health-related quality of life in chronic spinal cord injury. Am J Phys Med Rehabil. (2007) 86:387–96. 10.1097/PHM.0b013e31804a7d00 17449983 PMC2292343

[24] Anderson KD. Targeting recovery: priorities of the spinal cord-injured population. J Neurotrauma. (2004) 21:1371–83. 10.1089/neu.2004.21.1371 15672628

[25] New PW. Secondary conditions in a community sample of people with spinal cord damage. J Spinal Cord Med. (2016) 39:665–70. 10.1080/10790268.2016.1138600 26899984 PMC5137565

[26] Waites KB Canupp KC DeVivo MJ. Epidemiology and risk factors for urinary tract infection following spinal cord injury. Arch Phys Med Rehabili. (1993) 74:691–5. 10.1016/0003-9993(93)90026-7 8328888

[27] Norrbrink Budh C Lund I Hultling C Levi R Werhagen L Ertzgaard P. Gender related differences in pain in spinal cord injured individuals. Spinal Cord. (2003) 41:122–8. 10.1038/sj.sc.3101407 12595876

[28] Werhagen L Budh CN Hultling C Molander C. Neuropathic pain after traumatic spinal cord injury–relations to gender, spinal level, completeness, and age at the time of injury. Spinal Cord. (2004) 42:665–73. 10.1038/sj.sc.3101641 15289801

[29] Dominguez CA Ström M Gao T Zhang L Olsson T Wiesenfeld-Hallin Z. Genetic and sex influence on neuropathic pain-like behaviour after spinal cord injury in the rat. Eur J Pain. (2012) 16:1368–77. 10.1002/j.1532-2149.2012.00144.x 22473909

[30] Hubscher CH Fell JD Gupta DS. Sex and hormonal variations in the development of at-level allodynia in a rat chronic spinal cord injury model. Neurosci Lett. (2010) 477:153–6. 10.1016/j.neulet.2010.04.053 20434524 PMC2883654

[31] Gensel J Donahue RR Bailey WM Taylor BK. Sexual dimorphism of pain control: analgesic effects of pioglitazone and azithromycin in chronic spinal cord injury. J Neurotrauma. (2019) 36:2372–6. 10.1089/neu.2018.6207 30618345 PMC6648167

[32] Park A Uddin O Li Y Masri R Keller A. Pain after spinal cord injury is associated with abnormal presynaptic inhibition in the posterior nucleus of the thalamus. J Pain. (2018) 19:727.e1–727.e15. 10.1016/j.jpain.2018.02.002 29481977 PMC6026070

[33] Cowie AM Dittel BN Stucky CL. A novel sex-dependent target for the treatment of postoperative pain: the NLRP3 inflammasome. Front Neurol. (2019) 10:622. 10.3389/fneur.2019.00622 31244767 PMC6581722

[34] Del Rivero T Fischer R Yang F Swanson KA Bethea JR Tumor necrosis factor receptor 1 inhibition is therapeutic for neuropathic pain in males but not in females. Pain. (2019) 160:922–31. 10.1097/j.pain.0000000000001470 30586024

[35] Fujita Y Yamada Y Kusama M Yamauchi T Kamon J Kadowaki T. Sex differences in the pharmacokinetics of pioglitazone in rats. Comp Biochem Physiol C Toxicol Pharmacol. (2003) 136:85–94. 10.1016/s1532-0456(03)00194-7 14522601

[36] Inyang KE Szabo-Pardi T Wentworth E McDougal TA Dussor G Burton MD The antidiabetic drug metformin prevents and reverses neuropathic pain and spinal cord microglial activation in male but not female mice. Pharmacol Res. (2019) 139:1–16. 10.1016/j.phrs.2018.10.027 30391353 PMC6447087

[37] Li L Fan X Warner M Xu X-J Gustafsson J-Å Wiesenfeld-Hallin Z. Ablation of estrogen receptor α or β eliminates sex differences in mechanical pain threshold in normal and inflamed mice. Pain. (2009) 143:37–40. 10.1016/j.pain.2009.01.005 19285805

[38] Noor S Sun MS Vanderwall AG Havard MA Sanchez JE Harris NW. LFA-1 antagonist. (BIRT377) similarly reverses peripheral neuropathic pain in male and female mice with underlying sex divergent peripheral immune proinflammatory phenotypes. Neuroimmunol Neuroinflamm. (2019) 6:10. 10.20517/2347-8659.2019.18 31763376 PMC6873931

[39] Stein DG Hoffman SW. Estrogen and progesterone as neuroprotective agents in the treatment of acute brain injuries. Pediatr Rehabil. (2003) 6:13–22. 10.1080/1363849031000095279 12745891

[40] Bramlett HM Dietrich WD Neuropathological protection after traumatic brain injury in intact female rats versus males or ovariectomized females. J Neurotrauma. (2001) 18:891–900. 10.1089/089771501750451811 11565601

[41] Swartz KR Fee DB Joy KM Roberts KN Sun S Scheff NN Gender differences in spinal cord injury are not estrogen-dependent. J Neurotrauma. (2007) 24:473–80. 10.1089/neu.2006.0167 17402853

[42] Aminmansour B Asnaashari A Rezvani M Ghaffarpasand F Amin Noorian SM Saboori M. Effects of progesterone and vitamin D on outcome of patients with acute traumatic spinal cord injury; a randomized, double-blind, placebo controlled study. J Spinal Cord Med. (2016) 39:272–80. 10.1080/10790268.2015.1114224 26832888 PMC5073761

[43] Elkabes S Nicot AB. Sex steroids and neuroprotection in spinal cord injury: a review of preclinical investigations. Exp Neurol. (2014) 259:28–37. 10.1016/j.expneurol.2014.01.008 24440641

[44] Garcia-Ovejero D González S Paniagua-Torija B Lima A Molina-Holgado E De Nicola AF. Progesterone reduces secondary damage, preserves white matter, and improves locomotor outcome after spinal cord contusion. J Neurotrauma. (2014) 31:857–71. 10.1089/neu.2013.3162 24460450 PMC3996974

[45] Lee JY Choi HY Ju B-G Yune TY. Estrogen alleviates neuropathic pain induced after spinal cord injury by inhibiting microglia and astrocyte activation. Biochim Biophys Acta Mol Basis Dis. (2018) 1864:2472–80. 10.1016/j.bbadis.2018.04.006 29653184

[46] Samandari R Hassanpour-Ezatti M Fakhri S Abbaszadeh F Jorjani M. Sex differences and role of gonadal hormones on glutamate levelafter spinal cord injury in rats: a microdialysis study. Basic Clin Neurosci. (2019) 10:225–34. 10.32598/bcn.9.10.260 31462977 PMC6712632

[47] Sengelaub DR Han Q Liu N-K Maczuga MA Szalavari V Valencia SA. Protective effects of estradiol and dihydrotestosterone following spinal cord injury. J Neurotrauma. (2018) 35:825–41. 10.1089/neu.2017.5329 29132243 PMC5863086

[48] Zendedel A Mönnink F Hassanzadeh G Zaminy A Ansar MM Habib P. Estrogen attenuates local inflammasome expression and activation after spinal cord injury. Mol Neurobiol. (2018) 55:1364–75. 10.1007/s12035-017-0400-2 28127698

[49] McCaughey EJ Purcell M McLean AN Fraser MH Bewick A Borotkanics RJ. Changing demographics of spinal cord injury over a 20-year period: a longitudinal population-based study in Scotland. Spinal Cord. (2016) 54:270–6. 10.1038/sc.2015.167 26458974 PMC5399148

[50] Doran SJ Ritzel RM Glaser EP Henry RJ Faden AI Loane DJ. Sex differences in acute neuroinflammation after experimental traumatic brain injury are mediated by infiltrating myeloid cells. J Neurotrauma. (2019) 36:1040–53. 10.1089/neu.2018.6019 30259790 PMC6444913

[51] Villapol S Loane DJ Burns MP. Sexual dimorphism in the inflammatory response to traumatic brain injury. Glia. (2017) 65:1423–38. 10.1002/glia.23171 28608978 PMC5609840

[52] Garland DE Adkins RH Stewart CA. Five-year longitudinal bone evaluations in individuals with chronic complete spinal cord injury. J Spinal Cord Med. (2008) 31:543–50. 10.1080/10790268.2008.11753650 19086712 PMC2607127

[53] Soldin OP Mattison DR. Sex differences in pharmacokinetics and pharmacodynamics. Clin Pharmacokinet. (2009) 48:143–57. 10.2165/00003088-200948030-00001 19385708 PMC3644551

[54] Arnold AP Breedlove SM. Organizational and activational effects of sex steroids on brain and behavior: a reanalysis. Horm Behav. (1985) 19:469–98. 10.1016/0018-506x(85)90042-x 3910535

[55] McCarthy MM. Sex differences in neuroimmunity as an inherent risk factor. Neuropsychopharmacology. (2019) 44:38–44. 10.1038/s41386-018-0138-1 29977075 PMC6235925

[56] Forger NG. The organizational hypothesis and final common pathways: sexual differentiation of the spinal cord and peripheral nervous system. Horm Behav. (2009) 55:605–10. 10.1016/j.yhbeh.2009.03.008 19446077 PMC2703449

[57] McCarthy MM Nugent BM. At the frontier of epigenetics of brain sex differences. Front Behav Neurosci. (2015) 9:221. 10.3389/fnbeh.2015.00221 26347630 PMC4543874

[58] Morris JA Jordan CL Breedlove SM. Sexual differentiation of the vertebrate nervous system. Nat Neurosci. (2004) 7:1034–9. 10.1038/nn1325 15452574

[59] Breedlove SM Arnold AP. Hormone accumulation in a sexually dimorphic motor nucleus of the rat spinal cord. Science. (1980) 210:564–6. 10.1126/science.7423210 7423210

[60] Holmes GM Sachs BD. Physiology and mechanics of rat levator ani muscle: evidence for a sexual function. Physiol Behav. (1994) 55:255–66. 10.1016/0031-9384(94)90131-7 8153163

[61] Forger NG Strahan JA Castillo-Ruiz A. Cellular and molecular mechanisms of sexual differentiation in the mammalian nervous system. Front Neuroendocrinol. (2016) 40:67–86. 10.1016/j.yfrne.2016.01.001 26790970 PMC4897775

[62] Mong JA McCarthy MM. Ontogeny of sexually dimorphic astrocytes in the neonatal rat arcuate. Brain Res Dev Brain Res. (2002) 139:151–8. 10.1016/s0165-3806(02)00541-2 12480129

[63] Villa A Gelosa P Castiglioni L Cimino M Rizzi N Pepe G. Sex-specific features of microglia from adult mice. Cell Rep. (2018) 23:3501–11. 10.1016/j.celrep.2018.05.048 29924994 PMC6024879

[64] Zhang B Gensel JC. Is neuroinflammation in the injured spinal cord different than in the brain? Examining intrinsic differences between the brain and spinal cord. Exp Neurol. (2014) 258:112–20. 10.1016/j.expneurol.2014.04.007 25017892

[65] Batchelor PE Tan S Wills TE Porritt MJ Howells DW. Comparison of inflammation in the brain and spinal cord following mechanical injury. J Neurotrauma. (2008) 25:1217–25. 10.1089/neu.2007.0308 18986223

[66] Schnell L Fearn S Klassen H Schwab ME Perry VH. Acute inflammatory responses to mechanical lesions in the CNS: differences between brain and spinal cord. Euro J Neurosci. (2008) 11:3648–58. 10.1046/j.1460-9568.1999.00792.x 10564372

[67] Baker AE Brautigam VM Watters JJ. Estrogen modulates microglial inflammatory mediator production via interactions with estrogen receptor beta. Endocrinology. (2004) 145:5021–32. 10.1210/en.2004-0619 15256495

[68] Smith JA Das A Butler JT Ray SK Banik NL Estrogen or estrogen receptor agonist inhibits lipopolysaccharide induced microglial activation and death. Neurochem Res. (2011) 36:1587–93. 10.1007/s11064-010-0336-7 21127968 PMC3951894

[69] Kipp M Beyer C. Impact of sex steroids on neuroinflammatory processes and experimental multiple sclerosis. Front Neuroendocrinol. (2009) 30:188–200. 10.1016/j.yfrne.2009.04.004 19393685

[70] Simpkins JW Wang J Wang X Perez E Prokai L Dykens JA. Mitochondria play a central role in estrogen-induced neuroprotection. Curr Drug Targets CNS Neurol Disord. (2005) 4:69–83. 10.2174/1568007053005073 15723615

[71] Wilson ME Liu Y Wise PM. Estradiol enhances Akt activation in cortical explant cultures following neuronal injury. Brain Res Mol Brain Res. (2002) 102:48–54. 10.1016/s0169-328x(02)00181-x 12191493

[72] Klinge CM. Estrogenic control of mitochondrial function and biogenesis. J Cell Biochem. (2008) 105:1342–51. 10.1002/jcb.21936 18846505 PMC2593138

[73] Monteiro R Teixeira D Calhau C. Estrogen signaling in metabolic inflammation. Media Inflamm. (2014) 2014:615917. 10.1155/2014/615917 25400333 PMC4226184

[74] Torres MJ Kew KA Ryan TE Pennington ER Lin C-T Buddo KA. 17β-Estradiol directly lowers mitochondrial membrane microviscosity and improves bioenergetic function in skeletal muscle. Cell Metabolism. (2018) 27:167–179.e7. 10.1016/j.cmet.2017.10.003 29103922 PMC5762397

[75] Devanney NA Stewart AN Gensel JC. Microglia and macrophage metabolism in CNS injury and disease: The role of immunometabolism in neurodegeneration and neurotrauma. Exp Neurol. (2020) 329:113310. 10.1016/j.expneurol.2020.113310 32289316 PMC7237336

[76] Ravi S Mitchell T Kramer PA Chacko B Darley-Usmar VM. Mitochondria in monocytes and macrophages-implications for translational and basic research. Int J Biochem Cell Biol. (2014) 53:202–7. 10.1016/j.biocel.2014.05.019 24863362 PMC4111987

[77] Kovats S. Estrogen receptors regulate innate immune cells and signaling pathways. Cell Immunol. (2015) 294:63–9. 10.1016/j.cellimm.2015.01.018 25682174 PMC4380804

[78] Wilson ME Dimayuga FO Reed JL Curry TE Anderson CF Nath A. Immune modulation by estrogens: role in CNS HIV-1 infection. Endocrine. (2006) 29:289–97. 10.1385/ENDO:29:2:289 16785604

[79] Pan MX Li J Ma C Fu K Li ZQ Wang ZF. Sex-dependent effects of GPER activation on neuroinflammation in a rat model of traumatic brain injury. Brain Behav Immun. (2020). 10.1016/j.bbi.2020.04.005. [Epub ahead of print]. 32272225

[80] Beagley KW Gockel CM. Regulation of innate and adaptive immunity by the female sex hormones oestradiol and progesterone. FEMS Immunol Med Microbiol. (2003) 38:13–22. 10.1016/S0928-8244(03)00202-5 12900050

[81] Klein SL Flanagan KL Sex differences in immune responses. Nat Rev Immunol. (2016) 16:626–38. 10.1038/nri.2016.90 27546235

[82] Straub RH. The complex role of estrogens in inflammation. Endocr Rev. (2007) 28:521–74. 10.1210/er.2007-0001 17640948

[83] Calippe B Douin-Echinard V Delpy L Laffargue M Lélu K Krust A. 17Beta-estradiol promotes TLR4-triggered proinflammatory mediator production through direct estrogen receptor alpha signaling in macrophages *in vivo*. J Immunol. (2010) 185:1169–76. 10.4049/jimmunol.0902383 20554954

[84] Rosen S Ham B Mogil JS. Sex differences in neuroimmunity and pain. J Neurosci Res. (2017) 95:500–8. 10.1002/jnr.23831 27870397

[85] Kverneland AH Streitz M Geissler E Hutchinson J Vogt K Boës D. Age and gender leucocytes variances and references values generated using the standardized ONE-Study protocol. Cytometry A. (2016) 89:543–64. 10.1002/cyto.a.22855 27144459

[86] Ngo ST Steyn FJ McCombe PA. Gender differences in autoimmune disease. Front Neuroendocrinol. (2014) 35:347–69. 10.1016/j.yfrne.2014.04.004 24793874

[87] Lin C-W Huang Y-P Pan S-L Spinal cord injury is related to an increased risk of multiple sclerosis: a population-based, propensity score-matched, longitudinal follow-up study. Journal of Neurotrauma. (2015) 32:655–9. 10.1089/neu.2014.3723 25545758 PMC4410755

[88] Ankeny DP Lucin KM Sanders VM McGaughy VM Popovich PG. Spinal cord injury triggers systemic autoimmunity: evidence for chronic B lymphocyte activation and lupus-like autoantibody synthesis. J Neurochem. (2006) 99:1073–87. 10.1111/j.1471-4159.2006.04147.x 17081140

[89] Kil K Zang YCQ Yang D Markowski J Fuoco GS Vendetti GC. T cell responses to myelin basic protein in patients with spinal cord injury and multiple sclerosis. J Neuroimmunol. (1999) 98:201–7. 10430053 10.1016/s0165-5728(99)00057-0

[90] Saltzman JW Battaglino RA Salles L Jha P Sudhakar S Garshick E. B-cell maturation antigen, a proliferation-inducing ligand, and B-cell activating factor are candidate mediators of spinal cord injury-induced autoimmunity. J Neurotrauma. (2013) 30:434–40. 10.1089/neu.2012.2501 23088438 PMC3627405

[91] Schwab JM Zhang Y Kopp MA Brommer B Popovich PG. The paradox of chronic neuroinflammation, systemic immune suppression, autoimmunity after traumatic chronic spinal cord injury. Exp Neurol. (2014) 258:121–9. 10.1016/j.expneurol.2014.04.023 25017893 PMC4099970

[92] Zajarías-Fainsod D Carrillo-Ruiz J Mestre H Grijalva I Madrazo I Ibarra A. Autoreactivity against myelin basic protein in patients with chronic paraplegia. Eur Spine J. (2012) 21:964–70. 10.1007/s00586-011-2060-7 22057439 PMC3337920

[93] Hauben E Agranov E Gothilf A Nevo U Cohen A Smirnov I. Posttraumatic therapeutic vaccination with modified myelin self-antigen prevents complete paralysis while avoiding autoimmune disease. J Clin Invest. (2001) 108:591–9. 10.1172/JCI12837 11518733 PMC209402

[94] Rettew JA Huet-Hudson YM Marriott I. Testosterone reduces macrophage expression in the mouse of toll-like receptor 4, a trigger for inflammation and innate immunity. Biol Reprod. (2008) 78:432–7. 18003947 10.1095/biolreprod.107.063545

[95] Ingersoll MA. Sex differences shape the response to infectious diseases. PLoS Pathog. (2017) 13:e1006688. 10.1371/journal.ppat.1006688 29284060 PMC5746274

[96] Steeg LG Klein SL SeXX matters in infectious disease pathogenesis. PLoS Pathog. (2016) 12:e1005374 10.1371/journal.ppat.1005374 26891052 PMC4759457

[97] Bianchi VE. The anti-inflammatory effects of testosterone. J Endocr Soc. (2018) 3:91–107. 10.1210/js.2018-00186 30582096 PMC6299269

[98] Kupelian V Hayes FJ Link CL Rosen R McKinlay JB. Inverse association of testosterone and the metabolic syndrome in men is consistent across race and ethnic groups. J Clin Endocrinol Metab. (2008) 93:3403–10. 10.1210/jc.2008-0054 18559915 PMC2567862

[99] Salam R Kshetrimayum AS Keisam R. Testosterone and metabolic syndrome: the link. Ind J Endocrinol Metab. (2012) 16(Suppl 1):S12–S19. 10.4103/2230-8210.94248 22701831 PMC3354945

[100] Gensel JC Popovich PG Controversies on the role of Inflammation in the injured spinal cord. In: Morganti-Kossmann C. Raghupathi R. Maas A, editors. Traumatic Brain and Spinal Cord Injury Challenges and Developments. New York, NY: Cambridge University Press (2012). p. 272–9. 10.1017/CBO9781139030564

[101] Hains BC Waxman SG. Activated microglia contribute to the maintenance of chronic pain after spinal cord injury. J Neurosci. (2006) 26:4308–17. 10.1523/JNEUROSCI.0003-06.2006 16624951 PMC6674010

[102] Chan W-M Mohammed Y Lee I Pearse DD. Effect of gender on recovery after spinal cord injury. Transl Stroke Res. (2013) 4:447–61. 10.1007/s12975-012-0249-7 24323341

[103] Caruso A Di Giorgi Gerevini V Castiglione M Marinelli F Tomassini V Pozzilli C. Testosterone amplifies excitotoxic damage of cultured oligodendrocytes. J Neurochem. (2004) 88:1179–85. 10.1046/j.1471-4159.2004.02284.x 15009673

[104] Cui R Kang Y Wang L Li S Ji X Yan W. Testosterone propionate exacerbates the deficits of nigrostriatal dopaminergic system and downregulates Nrf2 expression in reserpine-treated aged male rats. Front Aging Neurosci. (2017) 9:172. 10.3389/fnagi.2017.00172 28620296 PMC5449473

[105] Wang H Liu H Liu R-M. Gender difference in glutathione metabolism during aging in mice. Exp Gerontol. (2003) 38:507–17. 10.1016/s0531-5565(03)00036-6 12742528

[106] Liu H Harrell LE Shenvi S Hagen T Liu R-M. Gender differences in glutathione metabolism in Alzheimer's disease. J Neurosci Res. (2005) 79:861–7. 10.1002/jnr.20424 15693022

[107] Yokota K Kubota K Kobayakawa K Saito T Hara M Kijima K. Pathological changes of distal motor neurons after complete spinal cord injury. Mol Brain. (2019) 12:4. 10.1186/s13041-018-0422-3 30626449 PMC6327522

[108] Byers JS Huguenard AL Kuruppu D Liu N-K Xu X-M Sengelaub DR. Neuroprotective effects of testosterone on motoneuron and muscle morphology following spinal cord injury. J Comp Neurol. (2012) 520:2683–96. 10.1002/cne.23066 22314886 PMC3960947

[109] Otzel DM Lee J Ye F Borst SE Yarrow JF. Activity-based physical rehabilitation with adjuvant testosterone to promote neuromuscular recovery after spinal cord injury. Int J Mol Sci. (2018) 19:1701. 10.3390/ijms19061701 29880749 PMC6032131

[110] Griggs RB Donahue RR Morgenweck J Grace PM Sutton A Watkins LR. Pioglitazone rapidly reduces neuropathic pain through astrocyte and nongenomic PPARγ mechanisms. Pain. (2015) 156:469–82. 10.1097/01.j.pain.0000460333.79127 25599238 PMC4329091

[111] Bonofiglio D Gabriele S Aquila S Catalano S Gentile M Middea E. Estrogen receptor alpha binds to peroxisome proliferator-activated receptor response element and negatively interferes with peroxisome proliferator-activated receptor gamma signaling in breast cancer cells. Clin Cancer Res. (2005) 11:6139–47. 10.1158/1078-0432.CCR-04-2453 16144913

[112] Foryst-Ludwig A Clemenz M Hohmann S Hartge M Sprang C Frost N. Metabolic actions of estrogen receptor beta. (ERbeta) are mediated by a negative cross-talk with PPARgamma. PLoS Genet. (2008) 4:e1000108. 10.1371/journal.pgen.1000108 18584035 PMC2432036

[113] Keller H Givel F Perroud M Wahli W. Signaling cross-talk between peroxisome proliferator-activated receptor/retinoid X receptor and estrogen receptor through estrogen response elements. Mol Endocrinol. (1995) 9:794–804. 10.1210/mend.9.7.7476963 7476963

[114] Yoon M. PPARα in obesity: sex difference and estrogen involvement. PPAR Res. (2010) 2010:584296. 10.1155/2010/584296 20871824 PMC2943125

[115] Campbell SE Mehan KA Tunstall RJ Febbraio MA Cameron-Smith D. 17beta-estradiol upregulates the expression of peroxisome proliferator-activated receptor alpha and lipid oxidative genes in skeletal muscle. J Mol Endocrinol. (2003) 31:37–45. 10.1677/jme.0.0310037 12914523

[116] Jalouli M Carlsson L Améen C Lindén D Ljungberg A Michalik L. sex difference in hepatic peroxisome proliferator-activated receptor α expression: influence of pituitary and gonadal hormones. Endocrinology. (2003) 144:101–9. 10.1210/en.2002-220630 12488335

[117] Kadowaki K Fukino K Negishi E Ueno K. Sex differences in PPARgamma expressions in rat adipose tissues. Biol Pharm Bull. (2007) 30:818–20. 10.1248/bpb.30.818 17409529

[118] Park H-J Choi J-M. Sex-specific regulation of immune responses by PPARs. Exp Mol Med. (2017) 49:e364. 10.1038/emm.2017.102 28775365 PMC5579504

[119] Liu Y Park J-M Chang K-H Huh HJ Lee K Lee M-Y. AMP-Activated protein kinase mediates the antiplatelet effects of the thiazolidinediones rosiglitazone and pioglitazone. Mol Pharmacol. (2016) 89:313. 10.1124/mol.115.102004 26643379

[120] Papageorgiou E Pitulis N Msaouel P Lembessis P Koutsilieris M. The non-genomic crosstalk between PPAR-γ ligands and ERK1/2 in cancer cell lines. Exp Opin Therap Targets. (2007) 11:1071–85. 10.1517/14728222.11.8.1071 17665979

[121] Soldin OP Chung SH Mattison DR. Sex differences in drug disposition. J Biomed Biotechnol. (2011) 2011:187103. 10.1155/2011/187103 21403873 PMC3051160

[122] Nicolas J-M Espie P Molimard M. Gender and interindividual variability in pharmacokinetics. Drug Metab Rev. (2009) 41:408–21. 10.1080/10837450902891485 19601720

[123] Fenn AM Hall JCE Gensel JC Popovich PG Godbout JP IL-4 signaling drives a unique arginase+/IL-1 + microglia phenotype and recruits macrophages to the inflammatory CNS: consequences of age-related deficits in IL-4R after traumatic spinal cord injury. J Neurosci. (2014) 34:8904–17. 10.1523/JNEUROSCI.1146-14.2014 24966389 PMC4069360

[124] Geoffroy CG Hilton BJ Tetzlaff W Zheng B. Evidence for an age-dependent decline in axon regeneration in the adult mammalian central nervous system. Cell Rep. (2016) 15:238–46. 10.1016/j.celrep.2016.03.028 27050519 PMC5050004

[125] Geoffroy CG Meves JM Zheng B. The age factor in axonal repair after spinal cord injury: A focus on neuron-intrinsic mechanisms. Neurosci Lett. (2017) 652:41–9. 10.1016/j.neulet.2016.11.003 27818358 PMC5415436

[126] Leden RE Khayrullina G Moritz KE Byrnes KR Age exacerbates microglial activation, oxidative stress, inflammatory and NOX2 gene expression, and delays functional recovery in a middle-aged rodent model of spinal cord injury. J Neuroinflamm. (2017) 14:1–4. 10.1186/s12974-017-0933-3 PMC5563003 28821269

[127] Siegenthaler MM Berchtold NC Cotman CW Keirstead HS. Voluntary running attenuates age-related deficits following SCI. Exp Neurol. (2008) 210:207–16. 10.1016/j.expneurol.2007.10.019 18164294 PMC2387276

[128] Zhang B Bailey WM Braun KJ Gensel JC. Age decreases macrophage IL-10 expression: implications for functional recovery and tissue repair in spinal cord injury. Exp Neurol. (2015) 273:83–91. 10.1016/j.expneurol.2015.08.001 26263843 PMC4644435

[129] Varma A Hill EG Nicholas J Selassie A. Predictors of early mortality after traumatic spinal cord injury: a population-based study. Spine. (2010) 35:778–83. 10.1097/BRS.0b013e3181ba1359 20228715

[130] Barbonetti A Vassallo MRC Pacca F Cavallo F Costanzo M Felzani G. Correlates of low testosterone in men with chronic spinal cord injury. Andrology. (2014) 2:721–8. 10.1111/j.2047-2927.2014.00235.x 24925765

[131] Bauman WA La Fountaine MF Spungen AM. Age-related prevalence of low testosterone in men with spinal cord injury. J Spinal Cord Med. (2014) 37:32–9. 10.1179/2045772313Y.0000000122 24090163 PMC4066549

[132] Vermeulen A Goemaere S Kaufman JM. Testosterone, body composition and aging. J Endocrinol Invest. (1999) 22:110–6. 10442580

[133] Bachman E Travison TG Basaria S Davda MN Guo W Li M. Testosterone induces erythrocytosis via increased erythropoietin and suppressed hepcidin: evidence for a new erythropoietin/hemoglobin set point. J Gerontol A Biol Sci Med Sci. (2014) 69:725–35. 10.1093/gerona/glt154 24158761 PMC4022090

[134] Rochira V Zirilli L Madeo B Maffei L Carani C Testosterone action on erythropoiesis does not require its aromatization to estrogen: Insights from the testosterone and estrogen treatment of two aromatase-deficient men. J Steroid Biochem Mol Biol. (2009) 113:189–94. 10.1016/j.jsbmb.2008.12.007 19159688

[135] Guo W Li M Bhasin S. Testosterone supplementation improves anemia in aging male mice. J Gerontol A Biol Sci Med Sci. (2014) 69:505–13. 10.1093/gerona/glt127 23974081 PMC3991143

[136] Gerendai I Banczerowski P Halász B. Functional significance of the innervation of the gonads. Endocrine. (2005) 28:309–18. 10.1385/ENDO:28:3:309 16388121

[137] Breen KM Karsch FJ. New insights regarding glucocorticoids, stress and gonadotropin suppression. Front Neuroendocrinol. (2006) 27:233–45. 10.1016/j.yfrne.2006.03.335 16712908

[138] Michael AE Pester LA Curtis P Shaw RW Edwards CR Cooke BA. Direct inhibition of ovarian steroidogenesis by cortisol and the modulatory role of 11 beta-hydroxysteroid dehydrogenase. Clin Endocrinol. (1993) 38:641–4. 10.1111/j.1365-2265.1993.tb02147.x 8334750

[139] Ycaza Herrera A Mather M. Actions and interactions of estradiol and glucocorticoids in cognition and the brain: Implications for aging women. Neurosci Biobehav Rev. (2015) 55:36–52. 10.1016/j.neubiorev.2015.04.005 25929443 PMC4501869

[140] Sameni HR Yousefi B Effect of spinal cord injury on ovarian histomorphometric structure in rats. Iran J Basic Med Sci. (2003) 6:132–8.

[141] Zarbakhsh S Tabrizi Amjad M Yousefi B Aldaghi M Sameni H Histopathological and follicular atresia assessment of rat's ovarian tissue following experimental chronic spinal cord injury. Middle East J Rehabilit Health Studies. (2017) 4:e14303 10.5812/mejrh.14303

[142] Shah PK Song J Kim S Zhong H Roy RR Edgerton VR. Rodent estrous cycle response to incomplete spinal cord injury, surgical interventions, and locomotor training. Behav Neurosci. (2011) 125:996–1002. 10.1037/a0026032 22122153 PMC3361964

[143] Shunmugavel A Khan M Chou PC-T Singh I. Spinal cord injury induced arrest in estrous cycle of rats is ameliorated by S-nitrosoglutathione: novel therapeutic agent to treat amenorrhea. J Sex Med. (2012) 9:148–58. 10.1111/j.1743-6109.2011.02526.x 22024253 PMC3809072

[144] Goldman JM Murr AS Cooper RL. The rodent estrous cycle: characterization of vaginal cytology and its utility in toxicological studies. Birth Defects Res B Dev Reprod Toxicol. (2007) 80:84–97. 10.1002/bdrb.20106 17342777

[145] Goldmann T Wieghofer P Müller PF Wolf Y Varol D Yona S. A new type of microglia gene targeting shows TAK1 to be pivotal in CNS autoimmune inflammation. Nat Neurosci. (2013) 16:1618–26. 10.1038/nn.3531 24077561

[146] Goldstein D Zuckerman H Harpaz S Barkai J Geva A Gordon S. Correlation between estradiol and progesterone in cycles with luteal phase deficiency. Fertil Steril. (1982) 37:348–54. 7060785

[147] Reed BG Carr BR The Normal Menstrual Cycle and the Control of Ovulation, In: Feingold KR, Anawalt B, Boyce A, et al., editors. Endotext [Internet]. South Dartmouth (MA): MDText.com, Inc (2000).

[148] Riggs BL. The mechanisms of estrogen regulation of bone resorption. J Clin Invest. (2000) 106:1203–4. 10.1172/JCI11468 11086020 PMC381441

[149] Bauman WA Spungen AM. Metabolic changes in persons after spinal cord injury. Phys Med Rehabil Clin N Am. (2000) 11:109–40. 10.1179/2045772314Y.0000000245 10680161

[150] Huang HF Linsenmeyer TA Li MT Giglio W Anesetti R Hagen J. Acute effects of spinal cord injury on the pituitary-testicular hormone axis and Sertoli cell functions: a time course study. J Androl. (1995) 16:148–57. 7559145

[151] Durga A Sepahpanah F Regozzi M Hastings J Crane DA. Prevalence of testosterone deficiency after spinal cord injury. Pmr. (2011) 3:929–32. 10.1016/j.pmrj.2011.07.008 22024324

[152] Kikuchi TA Skowsky WR El-Toraei I Swerdloff R. The pituitary-gonadal axis in spinal cord injury. Fertil Steril. (1976) 27:1142–5. 10.1016/s0015-0282(16)42130-8 971771

[153] Morley JE Distiller LA Lissoos I Lipschitz R Kay G Searle DL. Testicular function in patients with spinal cord damage. Horm Metab Res. (1979) 11:679–82. 10.1055/s-0028-1092799 395062

[154] Claus-Walker J Scurry M Carter RE Campos RJ. Steady state hormonal secretion in traumatic quadriplegia. J Clin Endocrinol Metab. (1977) 44:530–5. 10.1210/jcem-44-3-530 838851

[155] Lim CAR Nightingale TE Elliott S Krassioukov AV. Lifestyle modifications and pharmacological approaches to improve sexual function and satisfaction in men with spinal cord injury: a narrative review. Spinal Cord. (2019) 58:391–401. 10.1038/s41393-019-0404-z 31857687

[156] Cumming DC Quigley ME Yen SS. Acute suppression of circulating testosterone levels by cortisol in men. J Clin Endocrinol Metab. (1983) 57:671–3. 10.1210/jcem-57-3-671 6348068

[157] Clark MJ Petroski GF Mazurek MO Hagglund KJ Sherman AK Lammy AB. Testosterone replacement therapy and motor function in men with spinal cord injury: a retrospective analysis. Am J Phys Med Rehabil. (2008) 87:281–4. 10.1097/PHM.0b013e318168bbec 18356620

[158] Gorgey AS Abilmona SM Sima A Khalil RE Khan R Adler RA. A secondary analysis of testosterone and electrically evoked resistance training versus testosterone only. (TEREX-SCI) on untrained muscles after spinal cord injury: a pilot randomized clinical trial. Spinal Cord. (2020) 58:298–308. 10.1038/s41393-019-0364-3 31641203 PMC7065941

[159] Callahan A Anderson KD Beattie MS Bixby JL Ferguson AR Fouad K. Developing a data sharing community for spinal cord injury research. Exp Neurol. (2017) 295:135–43. 10.1016/j.expneurol.2017.05.012 28576567 PMC6448396

[160] Fouad K Bixby JL Callahan A Grethe JS Jakeman LB Lemmon VP. FAIR SCI ahead: the evolution of the open data commons for pre-clinical spinal cord injury research. J Neurotrauma. (2020) 37:831–8. 10.1089/neu.2019.6674 31608767 PMC7071068

[161] Hawryluk G Whetstone W Saigal R Ferguson A Talbott J Bresnahan J. Mean arterial blood pressure correlates with neurological recovery after human spinal cord injury: analysis of high frequency physiologic data. J Neurotrauma. (2015) 32:1958–67. 10.1089/neu.2014.3778 25669633 PMC4677564

[162] Hooshmand MJ Galvan MD Partida E Anderson AJ. Characterization of recovery, repair, and inflammatory processes following contusion spinal cord injury in old female rats: is age a limitation? Immun Ageing. (2014) 11:15. 10.1186/1742-4933-11-15 25512759 PMC4265993

[163] Berrocal Y Pearse DD Singh A Andrade CM McBroom JS Puentes R. Social and environmental enrichment improves sensory and motor recovery after severe contusive spinal cord injury in the rat. J Neurotrauma. (2007) 24:1761–72. 10.1089/neu.2007.0327 18001204

[164] Kappel S Hawkins P Mendl MT To group or not to group? Good practice for housing male laboratory mice. Animals. (2017) 7:88 10.3390/ani7120088 PMC5742782 29186765

[165] Basso DM Fisher LC Anderson AJ Jakeman LB McTigue DM Popovich PG. Basso Mouse Scale for locomotion detects differences in recovery after spinal cord injury in five common mouse strains. J Neurotrauma. (2006) 23:635–59. 10.1089/neu.2006.23.635 16689667

[166] Nieuwenhuis S Forstmann BU Wagenmakers E-J. Erroneous analyses of interactions in neuroscience: a problem of significance. Nat Neurosci. (2011) 14:1105–7. 10.1038/nn.2886 21878926

[167] Wilcox RR Tian T. Comparing dependent correlations. J Gen Psychol. (2008) 135:105–12. 10.3200/GENP.135.1.105-112 18318411

[168] Makin TR Orban de Xivry J-J Ten common statistical mistakes to watch out for when writing or reviewing a manuscript. Elife. (2019) 8:1 10.7554/eLife.48175 PMC6785265 31596231

