# Corrigendum: Considerations for Studying Sex as a Biological Variable in Spinal Cord Injury

**DOI:** 10.3389/fneur.2020.597689

**Published:** 2020-10-20

**Authors:** Andrew N. Stewart, Steven M. MacLean, Arnold J. Stromberg, Jessica P. Whelan, William M. Bailey, John C. Gensel, Melinda E. Wilson

**Affiliations:** ^1^Department of Physiology, University of Kentucky, Lexington, KY, United States; ^2^Spinal Cord and Brain Injury Research Center, College of Medicine, University of Kentucky, Lexington, KY, United States; ^3^Department of Statistics, College of Arts and Sciences, University of Kentucky, Lexington, KY, United States

**Keywords:** gender, stroke, traumatic brain injury (TBI), estrogen, testosterone, bladder, pain

In the original article, there was a mistake in the legend for Figure 3A as published. Specifically, the data code for a few animals in the original analysis in Figure 3A was incorrect. The correct legend appears below.

**Figure 3:**
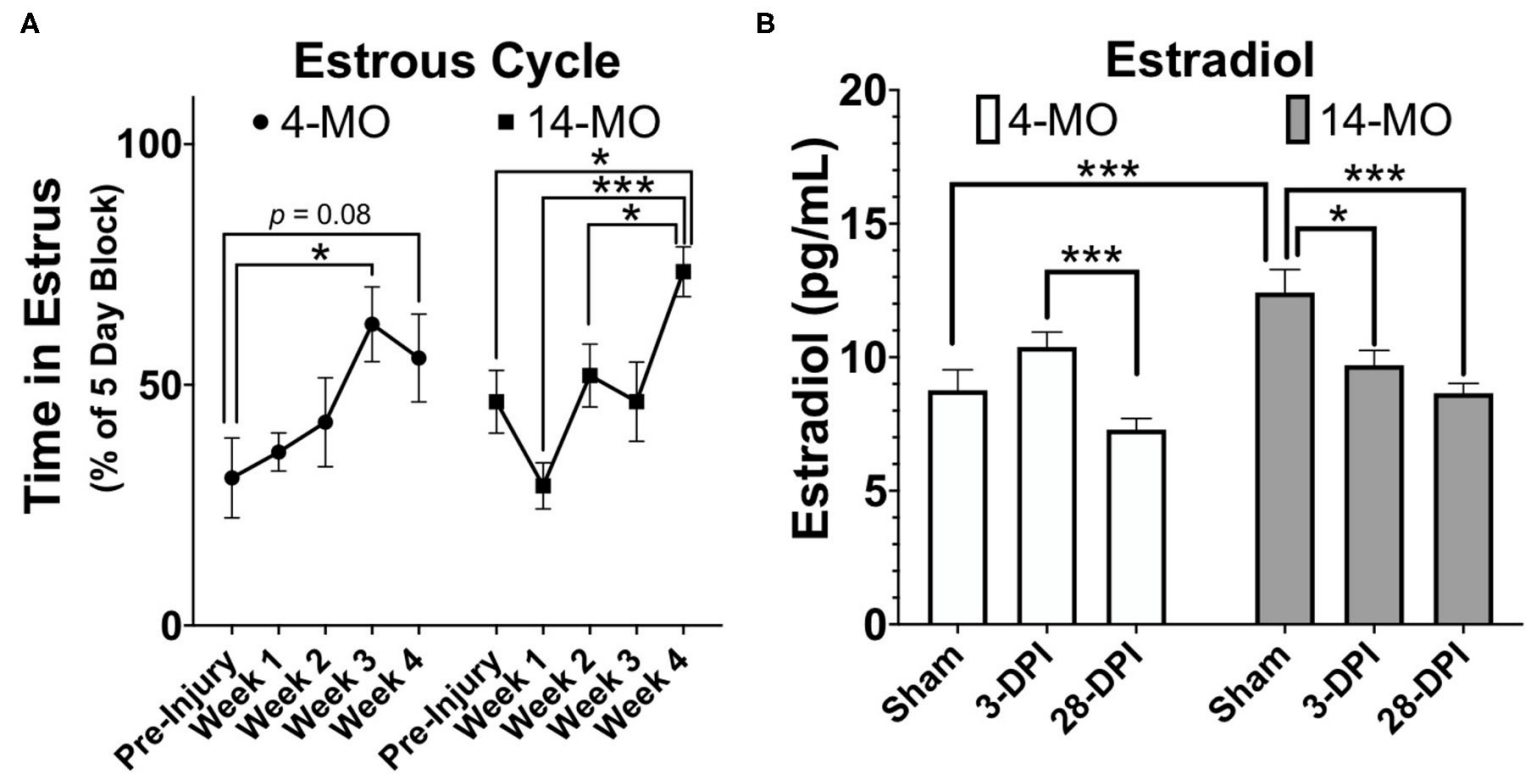
SCI (60 kDyn contusion) induces estrus cycle dysfunction **(A)** concurrent with decreased plasma estradiol by 28-DPI **(B)** in C57Bl/6J female mice. **(A)** Estrus cycle monitoring was performed for 28-DPI throughout the week and analyzed for estrous stage as previously described (144). Percent of time spent in estrous throughout a consistent 5-day block each week was assessed and used for analysis. Two-way repeated-measures ANOVA was used for analyses and revealed a significant main effect of time (*p* < 0.001) with both 4- and 14-MO mice demonstrating a significant increase in time spent in estrous compared to pre-injury conditions (*p* < 0.05; *n* = 9–10). Mean ± SEM. *p < 0.05; **p < 0.01, ***p < 0.001.

In the original article, there was an error. Because of the error in the coding of the animals (above) there was an error in the description of Figure 3A.

A correction has been made to *SCI Induces Estrous Cycle Dysfunction and Reduces Estradiol*, ***Paragraph 2***:

SCI dysregulates estrous cycling in rats, resulting in prolonged cycle duration (142, 143). By blocking time into week intervals post-SCI, we have found similar results in mice that SCI expands time spent in the estrous phase of the cycle [*F*_4, 36_ = 6.74, *p* < 0.001; Figure 3A] with a significant increase found by 28-DPI (*p* < 0.001) compared to pre-injury levels when age is combined. When comparing within an age, 4-MO mice reached a significant increase in time spent in the estrous phase compared to pre-injury levels by 21-DPI (*p* < 0.05) and 14-MO mice reached significance by 28-DPI (*p* < 0.05). Correspondingly, we also found a time by age interaction [*F*_2, 33_ = 6.08, *p* < 0.01; Figure 3B] in the plasma estradiol response to SCI likely owing to a modest increase in estradiol in 4-MO-, but a significant decrease in 14-MO female mice at 3-DPI (*p* < 0.05). Only 14-MO mice had a significant decrease in plasma estradiol levels at 28 days post-SCI compared to pre-injury values (*p* < 0.001). An inverse relationship between increased cycle duration and decreased estradiol is compatible with hormonal feedback mechanisms. Estrogens increases during pro-estrus until critical concentrations trigger an LH surge and ovulation, facilitating a transition into estrus. Therefore, decreased plasma estradiol will result in prolonged cycle duration which may delay the onset of an LH surge (145–147).”

The authors apologize for this error and state that this does not change the scientific conclusions of the article in any way. The original article has been updated.

