# Supplementary material for: Considerations for Studying Sex as a Biological Variable in Spinal Cord Injury
Source: Front Neurol. 2020 Aug 5;11:802. doi: 10.3389/fneur.2020.00802 (PMC7419700; doi:10.3389/fneur.2020.00802)
Supplement: Supplementary file 1 [file Table_1.DOCX]

| **Supplementary Table 1: 2018 SCI Pre-Clinical Research Use of Sex as a Biological Variable** | | | | | |
| --- | --- | --- | --- | --- | --- |
| **Author** | | | **Species** | **Sex** | **Citation** |
| Faw | et al., | 2018 | Mice | Both | Faw, T. D., Lerch, J. K., Thaxton, T. T., Deibert, R. J., Fisher, L. C., & Basso, D. M. (2018). Unique Sensory and Motor Behavior in Thy1-GFP-M Mice before and after Spinal Cord Injury. *Journal of neurotrauma*, *35*(18), 2167–2182. https://doi.org/10.1089/neu.2017.5395 |
| Wang | et al., | 2018 | Mice | Both | Wang, Y., Wu, W., Wu, X., Sun, Y., Zhang, Y. P., Deng, L. X., Walker, M. J., Qu, W., Chen, C., Liu, N. K., Han, Q., Dai, H., Shields, L. B., Shields, C. B., Sengelaub, D. R., Jones, K. J., Smith, G. M., & Xu, X. M. (2018). Remodeling of lumbar motor circuitry remote to a thoracic spinal cord injury promotes locomotor recovery. *eLife*, *7*, e39016. https://doi.org/10.7554/eLife.39016 |
| Cooper | et al., | 2018 | Mice | Both | Cooper, J. G., Jeong, S. J., McGuire, T. L., Sharma, S., Wang, W., Bhattacharyya, S., Varga, J., & Kessler, J. A. (2018). Fibronectin EDA forms the chronic fibrotic scar after contusive spinal cord injury. *Neurobiology of disease*, *116*, 60–68. https://doi.org/10.1016/j.nbd.2018.04.014 |
| Park | et al., | 2018 | Mice | Both | Park, A., Uddin, O., Li, Y., Masri, R., & Keller, A. (2018). Pain After Spinal Cord Injury Is Associated With Abnormal Presynaptic Inhibition in the Posterior Nucleus of the Thalamus. *The journal of pain : official journal of the American Pain Society*, *19*(7), 727.e1–727.e15. https://doi.org/10.1016/j.jpain.2018.02.002 |
| Hesp | et al., | 2018 | Mice | Both | Hesp, Z. C., Yoseph, R. Y., Suzuki, R., Jukkola, P., Wilson, C., Nishiyama, A., & McTigue, D. M. (2018). Proliferating NG2-Cell-Dependent Angiogenesis and Scar Formation Alter Axon Growth and Functional Recovery After Spinal Cord Injury in Mice. *The Journal of neuroscience : the official journal of the Society for Neuroscience*, *38*(6), 1366–1382. https://doi.org/10.1523/JNEUROSCI.3953-16.2017 |
| Anderson | et al., | 2018 | Mice/ Rats | Both | Anderson, M. A., O'Shea, T. M., Burda, J. E., Ao, Y., Barlatey, S. L., Bernstein, A. M., Kim, J. H., James, N. D., Rogers, A., Kato, B., Wollenberg, A. L., Kawaguchi, R., Coppola, G., Wang, C., Deming, T. J., He, Z., Courtine, G., & Sofroniew, M. V. (2018). Required growth facilitators propel axon regeneration across complete spinal cord injury. *Nature*, *561*(7723), 396–400. https://doi.org/10.1038/s41586-018-0467-6 |
| Myers | et al., | 2018 | Mice | Female | Myers, S. A., Gobejishvili, L., Saraswat Ohri, S., Garrett Wilson, C., Andres, K. R., Riegler, A. S., Donde, H., Joshi-Barve, S., Barve, S., & Whittemore, S. R. (2019). Following spinal cord injury, PDE4B drives an acute, local inflammatory response and a chronic, systemic response exacerbated by gut dysbiosis and endotoxemia. *Neurobiology of disease*, *124*, 353–363. https://doi.org/10.1016/j.nbd.2018.12.008 |
| Zholudeva | et al., | 2018 | Rats | Female | Zholudeva, L. V., Iyer, N., Qiang, L., Spruance, V. M., Randelman, M. L., White, N. W., Bezdudnaya, T., Fischer, I., Sakiyama-Elbert, S. E., & Lane, M. A. (2018). Transplantation of Neural Progenitors and V2a Interneurons after Spinal Cord Injury. *Journal of neurotrauma*, *35*(24), 2883–2903. https://doi.org/10.1089/neu.2017.5439 |
| Goodus | et al., | 2018 | Rats | Female | Goodus, M. T., Sauerbeck, A. D., Popovich, P. G., Bruno, R. S., & McTigue, D. M. (2018). Dietary Green Tea Extract Prior to Spinal Cord Injury Prevents Hepatic Iron Overload but Does Not Improve Chronic Hepatic and Spinal Cord Pathology in Rats. *Journal of neurotrauma*, *35*(24), 2872–2882. https://doi.org/10.1089/neu.2018.5771 |
| Lopez-  Serrano | et al., | 2018 | Mice | Female | López-Serrano, C., Santos-Nogueira, E., Francos-Quijorna, I., Coll-Miró, M., Chun, J., & López-Vales, R. (2019). Lysophosphatidic acid receptor type 2 activation contributes to secondary damage after spinal cord injury in mice. *Brain, behavior, and immunity*, *76*, 258–267. https://doi.org/10.1016/j.bbi.2018.12.007 |
| Goulao | et al., | 2018 | Rats | Female | Goulão, M., Ghosh, B., Urban, M. W., Sahu, M., Mercogliano, C., Charsar, B. A., Komaravolu, S., Block, C. G., Smith, G. M., Wright, M. C., & Lepore, A. C. (2019). Astrocyte progenitor transplantation promotes regeneration of bulbospinal respiratory axons, recovery of diaphragm function, and a reduced macrophage response following cervical spinal cord injury. *Glia*, *67*(3), 452–466. https://doi.org/10.1002/glia.23555 |
| Warren | et al., | 2018 | Rats | Female | Warren, P. M., Steiger, S. C., Dick, T. E., MacFarlane, P. M., Alilain, W. J., & Silver, J. (2018). Rapid and robust restoration of breathing long after spinal cord injury. *Nature communications*, *9*(1), 4843. https://doi.org/10.1038/s41467-018-06937-0 |
| Zhang | et al., | 2018 | Mice | Female | Zhang, B., Bailey, W. M., McVicar, A. L., Stewart, A. N., Veldhorst, A. K., & Gensel, J. C. (2019). Reducing age-dependent monocyte-derived macrophage activation contributes to the therapeutic efficacy of NADPH oxidase inhibition in spinal cord injury. *Brain, behavior, and immunity*, *76*, 139–150. https://doi.org/10.1016/j.bbi.2018.11.013 |
| Dumont | et al., | 2018 | Mice | Female | Dumont, C. M., Munsell, M. K., Carlson, M. A., Cummings, B. J., Anderson, A. J., & Shea, L. D. (2018). Spinal Progenitor-Laden Bridges Support Earlier Axon Regeneration Following Spinal Cord Injury. *Tissue engineering. Part A*, *24*(21-22), 1588–1602. https://doi.org/10.1089/ten.TEA.2018.0053 |
| Smith | et al., | 2018 | Mice | Female | Smith, D. R., Margul, D. J., Dumont, C. M., Carlson, M. A., Munsell, M. K., Johnson, M., Cummings, B. J., Anderson, A. J., & Shea, L. D. (2019). Combinatorial lentiviral gene delivery of pro-oligodendrogenic factors for improving myelination of regenerating axons after spinal cord injury. *Biotechnology and bioengineering*, *116*(1), 155–167. https://doi.org/10.1002/bit.26838 |
| Salazar | et al., | 2018 | Rats | Female | Salazar, B. H., Hoffman, K. A., Zhang, C., Zhang, Y., Cruz, Y., Boone, T. B., & Munoz, A. (2019). Modulatory effects of intravesical P2X2/3 purinergic receptor inhibition on lower urinary tract electromyographic properties and voiding function of female rats with moderate or severe spinal cord injury. *BJU international*, *123*(3), 538–547. https://doi.org/10.1111/bju.14561 |
| Park | et al., | 2018 | Mice | Female | Park, J., Decker, J. T., Smith, D. R., Cummings, B. J., Anderson, A. J., & Shea, L. D. (2018). Reducing inflammation through delivery of lentivirus encoding for anti-inflammatory cytokines attenuates neuropathic pain after spinal cord injury. *Journal of controlled release : official journal of the Controlled Release Society*, *290*, 88–101. https://doi.org/10.1016/j.jconrel.2018.10.003 |
| Pearse | et al., | 2018 | Rats | Female | Pearse, D. D., Bastidas, J., Izabel, S. S., & Ghosh, M. (2018). Schwann Cell Transplantation Subdues the Pro-Inflammatory Innate Immune Cell Response after Spinal Cord Injury. *International journal of molecular sciences*, *19*(9), 2550. https://doi.org/10.3390/ijms19092550 |
| Wang | et al., | 2018 | Rats | Female | Wang, Q., Zhang, H., Xu, H., Zhao, Y., Li, Z., Li, J., Wang, H., Zhuge, D., Guo, X., Xu, H., Jones, S., Li, X., Jia, X., & Xiao, J. (2018). Novel multi-drug delivery hydrogel using scar-homing liposomes improves spinal cord injury repair. *Theranostics*, *8*(16), 4429–4446. https://doi.org/10.7150/thno.26717 |
| Gollihue | et al., | 2018 | Rats | Female | Gollihue, J. L., Patel, S. P., Eldahan, K. C., Cox, D. H., Donahue, R. R., Taylor, B. K., Sullivan, P. G., & Rabchevsky, A. G. (2018). Effects of Mitochondrial Transplantation on Bioenergetics, Cellular Incorporation, and Functional Recovery after Spinal Cord Injury. *Journal of neurotrauma*, *35*(15), 1800–1818. https://doi.org/10.1089/neu.2017.5605 |
| Patil | et al., | 2018 | Rats | Female | Patil, N., Truong, V., Holmberg, M. H., Lavoie, N. S., McCoy, M. R., Dutton, J. R., Holmberg, E. G., & Parr, A. M. (2018). Safety and Efficacy of Rose Bengal Derivatives for Glial Scar Ablation in Chronic Spinal Cord Injury. *Journal of neurotrauma*, *35*(15), 1745–1754. https://doi.org/10.1089/neu.2017.5398 |
| Lin | et al., | 2018 | Rats | Female | Lin, C. Y., Androjna, C., Rozic, R., Nguyen, B., Parsons, B., Midura, R. J., & Lee, Y. S. (2018). Differential Adaptations of the Musculoskeletal System after Spinal Cord Contusion and Transection in Rats. *Journal of neurotrauma*, *35*(15), 1737–1744. https://doi.org/10.1089/neu.2017.5444 |
| Thomton | et al., | 2018 | Rats | Female | Thornton, M. A., Mehta, M. D., Morad, T. T., Ingraham, K. L., Khankan, R. R., Griffis, K. G., Yeung, A. K., Zhong, H., Roy, R. R., Edgerton, V. R., & Phelps, P. E. (2018). Evidence of axon connectivity across a spinal cord transection in rats treated with epidural stimulation and motor training combined with olfactory ensheathing cell transplantation. *Experimental neurology*, *309*, 119–133. https://doi.org/10.1016/j.expneurol.2018.07.015 |
| Kullmann | et al., | 2018 | Mice | Female | Kullmann, F. A., Beckel, J. M., McDonnell, B., Gauthier, C., Lynn, A. M., Wolf-Johnston, A., Kanai, A., Zabbarova, I. V., Ikeda, Y., de Groat, W. C., & Birder, L. A. (2018). Involvement of TRPM4 in detrusor overactivity following spinal cord transection in mice. *Naunyn-Schmiedeberg's archives of pharmacology*, *391*(11), 1191–1202. https://doi.org/10.1007/s00210-018-1542-0 |
| Skinner | et al., | 2018 | Rats | Female | Skinner, N. P., Lee, S. Y., Kurpad, S. N., Schmit, B. D., Muftuler, L. T., & Budde, M. D. (2018). Filter-probe diffusion imaging improves spinal cord injury outcome prediction. *Annals of neurology*, *84*(1), 37–50. https://doi.org/10.1002/ana.25260 |
| Caponegro | et al., | 2018 | Mice | Female | Caponegro, M. D., Torres, L. F., Rastegar, C., Rath, N., Anderson, M. E., Robinson, J. K., & Tsirka, S. E. (2019). Pifithrin-μ modulates microglial activation and promotes histological recovery following spinal cord injury. *CNS neuroscience & therapeutics*, *25*(2), 200–214. https://doi.org/10.1111/cns.13000 |
| Shimizu | et al., | 2018 | Mice | Female | Shimizu, N., Wada, N., Shimizu, T., Suzuki, T., Takaoka, E. I., Kanai, A. J., de Groat, W. C., Hirayama, A., Hashimoto, M., Uemura, H., & Yoshimura, N. (2018). Effects of nerve growth factor neutralization on TRP channel expression in laser-captured bladder afferent neurons in mice with spinal cord injury. *Neuroscience letters*, *683*, 100–103. https://doi.org/10.1016/j.neulet.2018.06.049 |
| Saraswat | et al., | 2018 | Mice | Female | Saraswat Ohri, S., Bankston, A. N., Mullins, S. A., Liu, Y., Andres, K. R., Beare, J. E., Howard, R. M., Burke, D. A., Riegler, A. S., Smith, A. E., Hetman, M., & Whittemore, S. R. (2018). Blocking Autophagy in Oligodendrocytes Limits Functional Recovery after Spinal Cord Injury. *The Journal of neuroscience : the official journal of the Society for Neuroscience*, *38*(26), 5900–5912. https://doi.org/10.1523/JNEUROSCI.0679-17.2018 |
| Noristani | et al., | 2018 | Mice | Female | Noristani, H. N., They, L., & Perrin, F. E. (2018). C57BL/6 and Swiss Webster Mice Display Differences in Mobility, Gliosis, Microcavity Formation and Lesion Volume After Severe Spinal Cord Injury. *Frontiers in cellular neuroscience*, *12*, 173. https://doi.org/10.3389/fncel.2018.00173 |
| Cerqueira | et al., | 2018 | Rats | Female | Cerqueira, S. R., Lee, Y. S., Cornelison, R. C., Mertz, M. W., Wachs, R. A., Schmidt, C. E., & Bunge, M. B. (2018). Decellularized peripheral nerve supports Schwann cell transplants and axon growth following spinal cord injury. *Biomaterials*, *177*, 176–185. https://doi.org/10.1016/j.biomaterials.2018.05.049 |
| Zabbarova | et al., | 2018 | Mice | Female | Zabbarova, I. V., Ikeda, Y., Carder, E. J., Wipf, P., Wolf-Johnston, A. S., Birder, L. A., Yoshimura, N., Getchell, S. E., Almansoori, K., Tyagi, P., Fry, C. H., Drake, M. J., & Kanai, A. J. (2018). Targeting p75 neurotrophin receptors ameliorates spinal cord injury-induced detrusor sphincter dyssynergia in mice. *Neurourology and urodynamics*, *37*(8), 2452–2461. https://doi.org/10.1002/nau.23722 |
| Jo | et al., | 2018 | Rats | Female | Jo, M. J., Kumar, H., Joshi, H. P., Choi, H., Ko, W. K., Kim, J. M., Hwang, S., Park, S. Y., Sohn, S., Bello, A. B., Kim, K. T., Lee, S. H., Zeng, X., & Han, I. (2018). Oral Administration of α-Asarone Promotes Functional Recovery in Rats With Spinal Cord Injury. *Frontiers in pharmacology*, *9*, 445. https://doi.org/10.3389/fphar.2018.00445 |
| Brock | et al., | 2018 | Rats | Female | Brock, J. H., Graham, L., Staufenberg, E., Im, S., & Tuszynski, M. H. (2018). Rodent Neural Progenitor Cells Support Functional Recovery after Cervical Spinal Cord Contusion. *Journal of neurotrauma*, *35*(9), 1069–1078. https://doi.org/10.1089/neu.2017.5244 |
| Park | et al., | 2018 | Mice | Female | Park, J., Decker, J. T., Margul, D. J., Smith, D. R., Cummings, B. J., Anderson, A. J., & Shea, L. D. (2018). Local Immunomodulation with Anti-inflammatory Cytokine-Encoding Lentivirus Enhances Functional Recovery after Spinal Cord Injury. *Molecular therapy : the journal of the American Society of Gene Therapy*, *26*(7), 1756–1770. https://doi.org/10.1016/j.ymthe.2018.04.022 |
| Mironets | et al., | 2018 | Rats | Female | Mironets, E., Osei-Owusu, P., Bracchi-Ricard, V., Fischer, R., Owens, E. A., Ricard, J., Wu, D., Saltos, T., Collyer, E., Hou, S., Bethea, J. R., & Tom, V. J. (2018). Soluble TNFα Signaling within the Spinal Cord Contributes to the Development of Autonomic Dysreflexia and Ensuing Vascular and Immune Dysfunction after Spinal Cord Injury. *The Journal of neuroscience : the official journal of the Society for Neuroscience*, *38*(17), 4146–4162. https://doi.org/10.1523/JNEUROSCI.2376-17.2018 |
| Shimizu | et al., | 2018 | Mice | Female | Shimizu, T., Majima, T., Suzuki, T., Shimizu, N., Wada, N., Kadekawa, K., Takai, S., Takaoka, E., Kwon, J., Kanai, A. J., de Groat, W. C., Tyagi, P., Saito, M., & Yoshimura, N. (2018). Nerve growth factor-dependent hyperexcitability of capsaicin-sensitive bladder afferent neurones in mice with spinal cord injury. *Experimental physiology*, *103*(6), 896–904. https://doi.org/10.1113/EP086951 |
| Sekine | et al., | 2018 | Mice | Female | Sekine, Y., Siegel, C. S., Sekine-Konno, T., Cafferty, W., & Strittmatter, S. M. (2018). The nociceptin receptor inhibits axonal regeneration and recovery from spinal cord injury. *Science signaling*, *11*(524), eaao4180. https://doi.org/10.1126/scisignal.aao4180 |
| Geissler | et al., | 2018 | Rats | Female | Geissler, S. A., Sabin, A. L., Besser, R. R., Gooden, O. M., Shirk, B. D., Nguyen, Q. M., Khaing, Z. Z., & Schmidt, C. E. (2018). Biomimetic hydrogels direct spinal progenitor cell differentiation and promote functional recovery after spinal cord injury. *Journal of neural engineering*, *15*(2), 025004. https://doi.org/10.1088/1741-2552/aaa55c |
| Lee | et al., | 2018 | Rats | Female | Lee, Y. S., Funk, L. H., Lee, J. K., & Bunge, M. B. (2018). Macrophage depletion and Schwann cell transplantation reduce cyst size after rat contusive spinal cord injury. *Neural regeneration research*, *13*(4), 684–691. https://doi.org/10.4103/1673-5374.230295 |
| Harman | et al., | 2018 | Rats | Female | Harman, K. A., States, G., Wade, A., Stepp, C., Wainwright, G., DeVeau, K., King, N., Shum-Siu, A., & Magnuson, D. (2018). Temporal analysis of cardiovascular control and function following incomplete T3 and T10 spinal cord injury in rodents. *Physiological reports*, *6*(6), e13634. https://doi.org/10.14814/phy2.13634 |
| Bezdudnaya | et al., | 2018 | Rats | Female | Bezdudnaya, T., Hormigo, K. M., Marchenko, V., & Lane, M. A. (2018). Spontaneous respiratory plasticity following unilateral high cervical spinal cord injury in behaving rats. *Experimental neurology*, *305*, 56–65. https://doi.org/10.1016/j.expneurol.2018.03.014 |
| Ryu | et al., | 2018 | Mice | Female | Ryu, J. C., Tooke, K., Malley, S. E., Soulas, A., Weiss, T., Ganesh, N., Saidi, N., Daugherty, S., Saragovi, U., Ikeda, Y., Zabbarova, I., Kanai, A. J., Yoshiyama, M., Farhadi, H. F., de Groat, W. C., Vizzard, M. A., & Yoon, S. O. (2018). Role of proNGF/p75 signaling in bladder dysfunction after spinal cord injury. *The Journal of clinical investigation*, *128*(5), 1772–1786. https://doi.org/10.1172/JCI97837 |
| Eldahan | et al., | 2018 | Rats | Female | Eldahan, K. C., Cox, D. H., Gollihue, J. L., Patel, S. P., & Rabchevsky, A. G. (2018). Rapamycin Exacerbates Cardiovascular Dysfunction after Complete High-Thoracic Spinal Cord Injury. *Journal of neurotrauma*, *35*(6), 842–853. https://doi.org/10.1089/neu.2017.5184 |
| Sengelaub | et al., | 2018 | Rats | Female | Sengelaub, D. R., Han, Q., Liu, N. K., Maczuga, M. A., Szalavari, V., Valencia, S. A., & Xu, X. M. (2018). Protective Effects of Estradiol and Dihydrotestosterone following Spinal Cord Injury. *Journal of neurotrauma*, *35*(6), 825–841. https://doi.org/10.1089/neu.2017.5329 |
| Ganzer | et al., | 2018 | Rats | Female | Ganzer, P. D., Darrow, M. J., Meyers, E. C., Solorzano, B. R., Ruiz, A. D., Robertson, N. M., Adcock, K. S., James, J. T., Jeong, H. S., Becker, A. M., Goldberg, M. P., Pruitt, D. T., Hays, S. A., Kilgard, M. P., & Rennaker, R. L., 2nd (2018). Closed-loop neuromodulation restores network connectivity and motor control after spinal cord injury. *eLife*, *7*, e32058. https://doi.org/10.7554/eLife.32058 |
| Lin | et al., | 2018 | Mice | Female | Li, H., Kong, W., Chambers, C. R., Yu, D., Ganea, D., Tuma, R. F., & Ward, S. J. (2018). The non-psychoactive phytocannabinoid cannabidiol (CBD) attenuates pro-inflammatory mediators, T cell infiltration, and thermal sensitivity following spinal cord injury in mice. *Cellular immunology*, *329*, 1–9. https://doi.org/10.1016/j.cellimm.2018.02.016 |
| Wada | et al., | 2018 | Mice | Female | Wada, N., Shimizu, T., Shimizu, N., de Groat, W. C., Kanai, A. J., Tyagi, P., Kakizaki, H., & Yoshimura, N. (2018). The effect of neutralization of nerve growth factor (NGF) on bladder and urethral dysfunction in mice with spinal cord injury. *Neurourology and urodynamics*, *37*(6), 1889–1896. https://doi.org/10.1002/nau.23539 |
| Cordero | et al., | 2018 | Rats | Female | Cordero, K., Coronel, G. G., Serrano-Illán, M., Cruz-Bracero, J., Figueroa, J. D., & De León, M. (2018). Effects of Dietary Vitamin E Supplementation in Bladder Function and Spasticity during Spinal Cord Injury. *Brain sciences*, *8*(3), 38. https://doi.org/10.3390/brainsci8030038 |
| Blissett | et al., | 2018 | Rats | Female | Blissett, A. R., Deng, B., Wei, P., Walsh, K. J., Ollander, B., Sifford, J., Sauerbeck, A. D., McComb, D. W., McTigue, D. M., & Agarwal, G. (2018). Sub-cellular In-situ Characterization of Ferritin(iron) in a Rodent Model of Spinal Cord Injury. *Scientific reports*, *8*(1), 3567. https://doi.org/10.1038/s41598-018-21744-9 |
| Keller | et al., | 2018 | Rats | Female | Keller, A. V., Rees, K. M., Seibt, E. J., Wood, B. D., Wade, A. D., Morehouse, J., Shum-Siu, A., & Magnuson, D. (2018). Electromyographic patterns of the rat hindlimb in response to muscle stretch after spinal cord injury. *Spinal cord*, *56*(6), 560–568. https://doi.org/10.1038/s41393-018-0069-z |
| Thompson | et al., | 2018 | Rats | Female | Thompson, R. E., Pardieck, J., Smith, L., Kenny, P., Crawford, L., Shoichet, M., & Sakiyama-Elbert, S. (2018). Effect of hyaluronic acid hydrogels containing astrocyte-derived extracellular matrix and/or V2a interneurons on histologic outcomes following spinal cord injury. *Biomaterials*, *162*, 208–223. https://doi.org/10.1016/j.biomaterials.2018.02.013 |
| Springer | et al., | 2018 | Rats | Female | Springer, J. E., Visavadiya, N. P., Sullivan, P. G., & Hall, E. D. (2018). Post-Injury Treatment with NIM811 Promotes Recovery of Function in Adult Female Rats after Spinal Cord Contusion: A Dose-Response Study. *Journal of neurotrauma*, *35*(3), 492–499. https://doi.org/10.1089/neu.2017.5167 |
| Saraswat | et al., | 2018 | Mice | Female | Saraswat Ohri, S., Mullins, A., Hetman, M., & Whittemore, S. R. (2018). Activating Transcription Factor-6α Deletion Modulates the Endoplasmic Reticulum Stress Response after Spinal Cord Injury but Does Not Affect Locomotor Recovery. *Journal of neurotrauma*, *35*(3), 486–491. https://doi.org/10.1089/neu.2015.3993 |
| Falnikar | et al., | 2018 | Rats | Female | Falnikar, A., Stratton, J., Lin, R., Andrews, C. E., Tyburski, A., Trovillion, V. A., Gottschalk, C., Ghosh, B., Iacovitti, L., Elliott, M. B., & Lepore, A. C. (2018). Differential Response in Novel Stem Cell Niches of the Brain after Cervical Spinal Cord Injury and Traumatic Brain Injury. *Journal of neurotrauma*, *35*(18), 2195–2207. https://doi.org/10.1089/neu.2017.5497 |
| Turtle | et al., | 2018 | Rats | Male | Turtle, J. D., Henwood, M. K., Strain, M. M., Huang, Y. J., Miranda, R. C., & Grau, J. W. (2019). Engaging pain fibers after a spinal cord injury fosters hemorrhage and expands the area of secondary injury. *Experimental neurology*, *311*, 115–124. https://doi.org/10.1016/j.expneurol.2018.09.018 |
| McCreedy | et al., | 2018 | Mice | Male | McCreedy, D. A., Lee, S., Sontag, C. J., Weinstein, P., Olivas, A. D., Martinez, A. F., Fandel, T. M., Trivedi, A., Lowell, C. A., Rosen, S. D., & Noble-Haeusslein, L. J. (2018). Early Targeting of L-Selectin on Leukocytes Promotes Recovery after Spinal Cord Injury, Implicating Novel Mechanisms of Pathogenesis. *eNeuro*, *5*(4), ENEURO.0101-18.2018. https://doi.org/10.1523/ENEURO.0101-18.2018 |
| Khan | et al., | 2018 | Rats | Male | Khan, M., Dhammu, T. S., Singh, I., & Singh, A. K. (2018). Amelioration of spinal cord injury in rats by blocking peroxynitrite/calpain activity. *BMC neuroscience*, *19*(1), 50. https://doi.org/10.1186/s12868-018-0450-z |
| Turtle | et al., | 2018 | Rats | Male | Turtle, J. D., Strain, M. M., Reynolds, J. A., Huang, Y. J., Lee, K. H., Henwood, M. K., Garraway, S. M., & Grau, J. W. (2018). Pain Input After Spinal Cord Injury (SCI) Undermines Long-Term Recovery and Engages Signal Pathways That Promote Cell Death. *Frontiers in systems neuroscience*, *12*, 27. https://doi.org/10.3389/fnsys.2018.00027 |
| Bezdudnaya | et al., | 2018 | Rats | Male | Bezdudnaya, T., Lane, M. A., & Marchenko, V. (2018). Paced breathing and phrenic nerve responses evoked by epidural stimulation following complete high cervical spinal cord injury in rats. *Journal of applied physiology (Bethesda, Md. : 1985)*, *125*(3), 687–696. https://doi.org/10.1152/japplphysiol.00895.2017 |
| Huang | et al., | 2018 | Rats | Male | Huang, Y. J., & Grau, J. W. (2018). Ionic plasticity and pain: The loss of descending serotonergic fibers after spinal cord injury transforms how GABA affects pain. *Experimental neurology*, *306*, 105–116. https://doi.org/10.1016/j.expneurol.2018.05.002 |
| Page | et al., | 2018 | Rats | Male | Page, J. C., Park, J., Chen, Z., Cao, P., & Shi, R. (2018). Parallel Evaluation of Two Potassium Channel Blockers in Restoring Conduction in Mechanical Spinal Cord Injury in Rat. *Journal of neurotrauma*, *35*(9), 1057–1068. https://doi.org/10.1089/neu.2017.5297 |
| White | et al., | 2018 | Rats | Male | White, A. R., & Holmes, G. M. (2018). Anatomical and Functional Changes to the Colonic Neuromuscular Compartment after Experimental Spinal Cord Injury. *Journal of neurotrauma*, *35*(9), 1079–1090. https://doi.org/10.1089/neu.2017.5369 |
| Liu | et al., | 2018 | Mice | Male | Liu, S., Li, Y., Choi, H., Sarkar, C., Koh, E. Y., Wu, J., & Lipinski, M. M. (2018). Lysosomal damage after spinal cord injury causes accumulation of RIPK1 and RIPK3 proteins and potentiation of necroptosis. *Cell death & disease*, *9*(5), 476. https://doi.org/10.1038/s41419-018-0469-1 |
| Warren | et al., | 2018 | Rats | Male | Warren, P. M., Campanaro, C., Jacono, F. J., & Alilain, W. J. (2018). Mid-cervical spinal cord contusion causes robust deficits in respiratory parameters and pattern variability. *Experimental neurology*, *306*, 122–131. https://doi.org/10.1016/j.expneurol.2018.04.005 |
| Macks | et al., | 2018 | Rats | Male | Macks, C., Gwak, S. J., Lynn, M., & Lee, J. S. (2018). Rolipram-Loaded Polymeric Micelle Nanoparticle Reduces Secondary Injury after Rat Compression Spinal Cord Injury. *Journal of neurotrauma*, *35*(3), 582–592. https://doi.org/10.1089/neu.2017.5092 |
| Garcia | et al., | 2018 | Mice | Unreported | Garcia, V. B., Abbinanti, M. D., Harris-Warrick, R. M., & Schulz, D. J. (2018). Effects of Chronic Spinal Cord Injury on Relationships among Ion Channel and Receptor mRNAs in Mouse Lumbar Spinal Cord. *Neuroscience*, *393*, 42–60. https://doi.org/10.1016/j.neuroscience.2018.09.034 |
| Yamagami | et al., | 2018 | Mice | Unreported | Yamagami, T., Pleasure, D. E., Lam, K. S., & Zhou, C. J. (2018). Transient activation of Wnt/β-catenin signaling reporter in fibrotic scar formation after compression spinal cord injury in adult mice. *Biochemical and biophysical research communications*, *496*(4), 1302–1307. https://doi.org/10.1016/j.bbrc.2018.02.004 |
| **Search Terms Used in PMC** | | | (((rat) OR mouse)) AND ((((Spinal cord injury[Title]) OR spinal cord contusion[Title]) OR spinal cord transection[Title]) AND ( "2018/01/01"[PDat] : "2018/12/31"[PDat] )) | | |
