# Supplementary material for: Considerations for Studying Sex as a Biological Variable in Spinal Cord Injury
Source: Front Neurol. 2020 Aug 5;11:802. doi: 10.3389/fneur.2020.00802 (PMC7419700; doi:10.3389/fneur.2020.00802)
Supplement: Supplementary file 2 [file Table_2.DOCX]

**2 Materials and Methods**

***2.1 Spinal Cord Injury***

All procedures were approved by the University of Kentucky’s Institutional Animal Use and Care Committee. Male and Female C57Bl/6J mice of ages 4- (Jackson Laboratories) and 14-months old (MO; National Institute of Aging), received moderate contusion SCI at the T9 vertebra as previously described (60 kDyne; Infinite Horizons Impactor; (Gensel et al., 2019; Scheff et al., 2003))

. Post-surgery, animals received one subcutaneous injection of buprenorphine-SR (1 mg/kg) and one injection of antibiotic (5 mg/kg, enrofloxacin 2.27%: Norbrook Inc., Lenexa, KS, USA) in 2 ml of saline and were housed in warming cages overnight. Animals continued to receive antibiotic subcutaneously in 1 ml saline for 5 days. Mice received manual bladder expressions 2x/day for the duration of the study up to 28-days post-injury (DPI).

***2.2 Weight Loss and Mortality***

Weight loss data was compiled from a single study of mice surviving 28-DPI (Fig. 2A). Mice were weighed weekly for the 4-weeks of survival after SCI, and the peak weight loss occurring at 14-DPI was assessed between groups (n=9-10). Mortality was assessed over the course of two independent studies. All mice from each of four groups (4- and 14-MO, male and female) that survived the surgical procedures were analyzed for mortality prior to the scheduled euthanasia. Scheduled euthanasia dates included 3-, 7-, and 28-DPI. Only mice that died due to unknown reasons were used in analysis. Mice that died due to bladder rupture, surgical complications, or autophagy were not included in either the death count or total sample size. Mice that were found moribund during routine bladder care were euthanized prior to natural death and were included in analysis. Only 14-MO male mice were found moribund, other causes of death remain undetermined.

***2.3 Blood Collection and Analysis of Anemia and Estradiol***

At 3- or 28-DPI, animals were anesthetized and blood was collected through cardiac puncture into collection tubes containing EDTA (VWR BD367835). Blood was collected from sham conditions at 3-DPI, or after SCI at 3- or 28-DPI. Blood collection tubes were centrifuged to pellet the red blood cells (RBC) and plasma samples were sent to the University of Virginia’s Center for Research in Reproduction Ligand Assay and Analysis Core for analysis of estradiol. To measure crude RBC/Plasma ratios, a line was drawn at the interface between the RBCs and plasma, as well as at the top of the plasma on the tube after centrifugation but before sending for estradiol analysis. The proportion of RBC to plasma volume was measured with a ruler and a percentage was obtained. Data for RBC/Plasma ratios are presented at 28-DPI only because plasma samples for SCI-injured mice at 3-DPI were used for other purposes before making the observation that 14-MO males had less RBCs.

***2.4 Estrous Cycle Measurements***

For mice surviving 28-DPI, the estrous cycle was monitored as previously described (Goldman et al., 2007) for at least 1-week pre-SCI, and 4-weeks post-SCI. Briefly, a small drop of water was placed at the opening of the vagina and aspirated using a disposable pipette. The drop was placed onto a slide and allowed to dry before staining with crystal violet for imaging. The cellular morphology was characterized according to Goldman and colleagues (2007), to determine the state of the estrous cycle. The amount of time spent in the estrus stage of the cycle within the 5-days per week analyzed was determined and used for analysis.

***2.5 Locomotor Analysis with Single or Group Housing***

Locomotor recovery of mice after SCI was analyzed using the Basso Mouse Scale (BMS) as described previously, and data from two independent studies performed in our lab were compared (Basso et al., 2006; Orr and Gensel, 2017). Importantly, these studies were not performed at the same time, however, all injury parameters (60 kDyne at T9) were consistent between studies and all mice were 4-MO C57Bl/6J females (Jackson Laboratories). In one study, mice were housed in groups of 4-5, in the other, mice were single housed after SCI. Two experienced experimental raters who were blinded to the group in their respective studies were used for BMS assessments. Data obtained at 1- and 28-DPI were provided to compare locomotor outcomes.

***2.6 Statistics***

Two-way analysis of variance (ANOVA) with (Fig. 3A), or without (Fig 2A,C, Fig 3B, Fig 4), repeated measures was used to assess main effects of age and sex. Tukey’s (Fig. 2C, 3A,B, and 4), or Sidak’s (Fig 2A) pairwise comparisons were used when main effects reached a significant value of p < 0.05. Weight loss was assessed based on percentage of pre-SCI body weight. To assess effects of SCI on RBC/Plasma ratios, post-SCI values were normalized to values obtained from sham mice. All analyses were performed using Prism v 8.0 (Graphpad, San Diego, CA).

**Reference:**

Basso, D.M., Fisher, L.C., Anderson, A.J., Jakeman, L.B., McTigue, D.M., Popovich, P.G., 2006. Basso Mouse Scale for locomotion detects differences in recovery after spinal cord injury in five common mouse strains. Journal of Neurotrauma 23, 635–659. doi:10.1089/neu.2006.23.635

Gensel, J., Donahue, R.R., Bailey, W.M., Taylor, B.K., 2019. Sexual dimorphism of pain control: analgesic effects of pioglitazone and azithromycin in chronic spinal cord injury. Journal of Neurotrauma. doi:10.1089/neu.2018.6207

Goldman, J.M., Murr, A.S., Cooper, R.L., 2007. The rodent estrous cycle: characterization of vaginal cytology and its utility in toxicological studies. Birth Defects Res B Dev Reprod Toxicol 80, 84–97. doi:10.1002/bdrb.20106

Orr, M.B., Gensel, J.C., 2017. Interactions of primary insult biomechanics and secondary cascades in spinal cord injury: implications for therapy. Neural Regen Res 12, 1618–1619. doi:10.4103/1673-5374.217332

Scheff, S.W., Rabchevsky, A.G., Fugaccia, I., Main, J.A., Lumpp, J.E.J., 2003. Experimental modeling of spinal cord injury: characterization of a force-defined injury device. Journal of Neurotrauma 20, 179–193. doi:10.1089/08977150360547099
